# Leveraging genomic prediction to scan germplasm collection for crop improvement

**DOI:** 10.1371/journal.pone.0179191

**Published:** 2017-06-09

**Authors:** Leonardo de Azevedo Peixoto, Tara C. Moellers, Jiaoping Zhang, Aaron J. Lorenz, Leonardo L. Bhering, William D. Beavis, Asheesh K. Singh

**Affiliations:** 1 Department of Biology, Federal University of Viçosa, Viçosa, Minas Gerais, Brazil; 2 Department of Agronomy, Iowa State University, Ames, IA, United States of America; 3 Department of Agronomy and Plant Genetics, University of Minnesota, Minneapolis, MN, United States of America; University of Guelph, CANADA

## Abstract

The objective of this study was to explore the potential of genomic prediction (GP) for soybean resistance against *Sclerotinia sclerotiorum* (Lib.) de Bary, the causal agent of white mold (WM). A diverse panel of 465 soybean plant introduction accessions was phenotyped for WM resistance in replicated field and greenhouse tests. All plant accessions were previously genotyped using the SoySNP50K BeadChip. The predictive ability of six GP models were compared, and the impact of marker density and training population size on the predictive ability was investigated. Cross-prediction among environments was tested to determine the effectiveness of the prediction models. GP models had similar prediction accuracies for all experiments. Predictive ability did not improve significantly by using more than 5k SNPs, or by increasing the training population size (from 50% to 90% of the total of individuals). The GP model effectively predicted WM resistance across field and greenhouse experiments when each was used as either the training or validation population. The GP model was able to identify WM-resistant accessions in the USDA soybean germplasm collection that had previously been reported and were not included in the study panel. This study demonstrated the applicability of GP to identify useful genetic sources of WM resistance for soybean breeding. Further research will confirm the applicability of the proposed approach to other complex disease resistance traits and in other crops.

## Introduction

Sclerotinia stem rot or white mold (WM), caused by *Sclerotinia sclerotiorum* (Lib.) de Bary, is a devastating disease of many economically important crops including soybean [1]. Since its first report from Ontario, Canada [2], WM has become one of the major diseases impacting soybean production in the United States [3]. Current strategies, such as increased row spacing, reduced irrigation before and during crop flowering, and biocontrol, have not effectively controlled this disease [4]. Despite that fungicides reduce the WM disease severity and yield losses are reduced, the profit does not increase using fungicides [5]. Considering the low effectiveness for all of these strategies, the development of resistant cultivars remains an effective and economic approach to cope with WM [6].

Plant breeders have traditionally used specialized disease nurseries or indoor growth environments to determine the expression of resistance in cultivars. However, due to the low to moderate heritability of trait [7] and its vulnerability to environmental influences [8], phenotyping for WM resistance is time-consuming and costly. Several quantitative trait loci (QTL) associated with the disease’s resistance have been reported [9], demonstrating the quantitative inheritance of WM resistance in soybean. Although marker-assisted selection (MAS) has played an important role in soybean breeding for disease and pest resistance (e.g. soybean cyst nematode resistance [10], its application in the improvement of WM resistance is challenging because WM resistance is controlled by many loci with small effects.

Genomic prediction entails building a prediction model by associating marker information with phenotypic information in a model training step [11]. Individual genetic material(s) that have been genotyped and phenotyped comprise the training population. The prediction model is then applied to a set of selection candidates that have been genotyped but not evaluated phenotypically (validation population). The primary difference between GP and traditional forms of MAS is that GP foregoes QTL identification through statistical significance tests and testing of significant markers by modeling all scored markers simultaneously. By utilizing genome-wide molecular markers, GP is becoming a promising method for the selection of complex traits in plant breeding programs [12] and has been applied to multiple crops including wheat, maize, and barley [13–15]. The prediction accuracies of GP have been reported to be 28% greater than some MAS and 95% as accurate as phenotypic selection for a single trait in wheat [16].

Relatively few GP studies have been reported in soybean [17–20] for quantitative traits including seed yield [17], seed weight [20], resistance to sudden death syndrome [21] and soybean cyst nematode [18]. However, the efficiency of GP on WM resistance, a complex trait in soybean, is currently unclear, making it an ideal trait to examine the efficiency of GP on soybean complex traits.

The effectiveness of GP depends on the correlation between the predicted genotypic value and the underlying true genotypic value [22]. This correlation, also called prediction accuracy, of GP has been expressed as a function of the training population size (TPS), trait heritability on an entry-mean basis (h^2^), and marker density [23,24]. Simulation and cross-validation studies have indicated that prediction accuracy generally increases as more individuals are included in the training population [16,25–30] and more markers were used [31]. However, when genome wide selection (GWS) is applied in a structured population, increasing the number of markers or the number of individuals of training population did not necessary lead to an increase of prediction accuracy [32]. Therefore, the first steps are to determine the training population size and number of markers to use for GP of genomic estimated breeding value (GEBV), and the appropriate prediction model to use to obtain high predictive ability, the correlation between the predicted breeding values and the observed phenotypic values as true breeding values are unknown in real datasets. The next steps are to validate the models and testing them to predict diverse accessions previously unseen in the training models. These efforts will not only provide information on the applicability of these prediction models but also complement the efforts to diversify the genetic basis of commercial soybean breeding programs by identifying and utilization of diverse accessions to broaden the genetic base of disease resistance.

Here we report the results of performing GP cross-validation for WM resistance from a collection of 465 plant introduction (PI) soybean lines from the USDA soybean germplasm collection. For the GP models, the training population composition, marker number, and the statistical method for the calculation of GEBV were varied to determine their effect on WM GP accuracy. To assess GP accuracy, cross validation was done to predict white mold (WM) in the field and greenhouse in 2014 and 2015. Finally, using the GP models, we validated previously reported sources of WM resistance as well as identified new sources of WM resistance in the entire USDA soybean germplasm collection, which houses 19,652 accessions from several geographical origins.

## Results

### Phenotypic evaluation and GP method comparison

**Fig 1:**
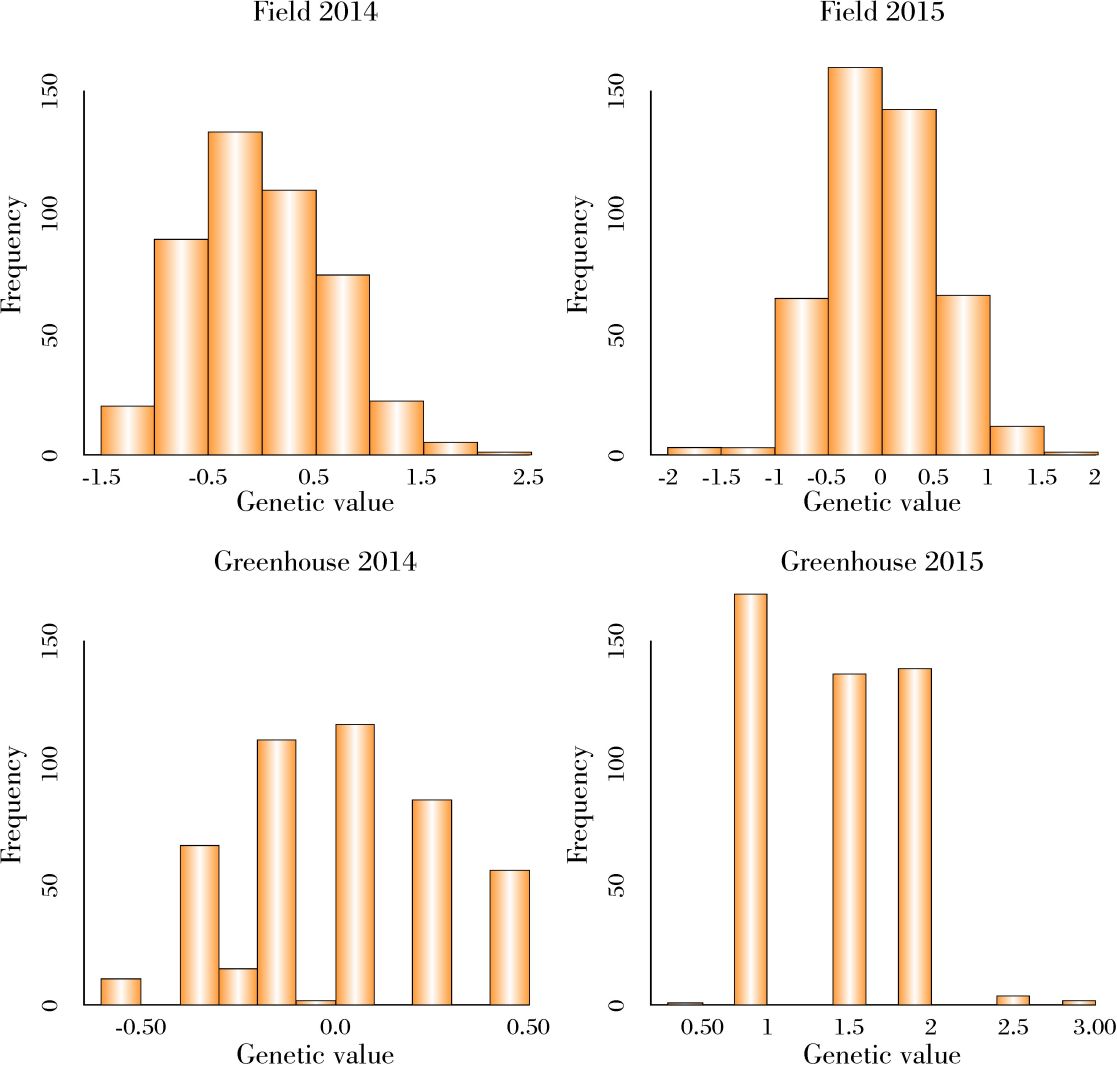
White mold phenotypic data distribution transformed using logistic regression of 465 soybean accessions tested in field and greenhouse specialized tests.

Continuous distribution was observed in the field experiments, asymmetric distribution was observed in GH2014, and a kurtotic distribution was observed in GH2015 based on the predicted value of the accession random effects in the logistic model (Fig 1). Asymmetric distribution was observed to Field2014, Field2015 and GH2014 and normal distribution to GH2015 for original data (S1 Fig). Few resistant accessions (score = 0) in the greenhouse experiments and several resistant accessions in the field experiments were observed (S1 Fig). The PI lines were considered resistant when their predicted value was low or equal compared with the resistant checks.

High genotypic correlation was observed between all experiments (S2 Fig). The genetic correlation between Field2014 and Field2015 was the highest (cor = 0.72) while the lowest correlation was between GH2014 and GH2015 (cor = 0.46). Correlations between field and greenhouse were almost the same ranging from 0.53 (Field2014 and GH2015) to 0.54 (Field2014 and GH2014, Field2015 and GH2014, Field2015 and GH2015).

The broad-sense heritability estimate for disease severity index (DSI) was 0.64 across field environments, suggesting that selections done for white mold resistance in a field setting would be effective. Genetic diversity estimated by principal component analysis (PCA) showed the diversity in the mini-core panel used in this study, and no pattern among PI accessions was detected (S3 Fig). When applying PCA to the genomic relationship matrix, 141 components of a total of 465 were needed to explain 80% of the total variance of genotypes. The first component captured 15.41% of the total variation of marker genotypes, whereas the second component explained 6.66% of the total variation (S3 Fig).

All GP methods tested in this study had similar prediction abilities of WM resistance in soybean, and these ranged from 0.43 to 0.47 in the field and from 0.18 to 0.25 in greenhouse testing (S4 Fig). The predictive ability from the field experiments was approximately twice that of the greenhouse experiments using the same model.

### Effect of marker density and training population size in the genomic prediction

**Fig 2:**
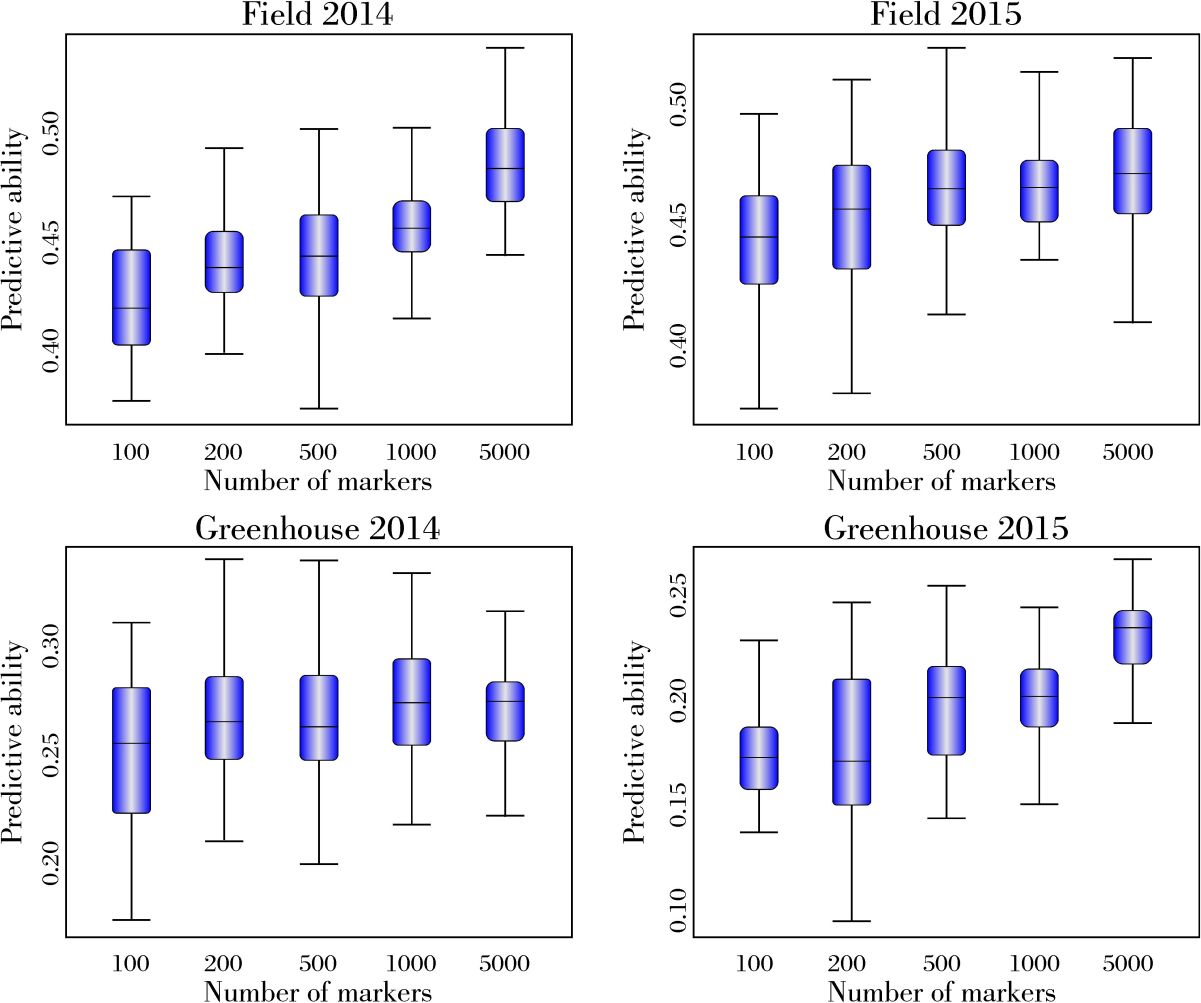
Relationship between predictive ability and the number of SNP markers using a training population of 352 genotypes among 465 diverse soybean accessions tested for white mold in 2014 and 2015 field and greenhouse experiments.

For all experiments and training population sizes, minimal increase was observed in the predictive ability for a given training population size beyond 5k SNPs (Fig 2). The increase for prediction ability comparing 100 markers and 5k markers was 14%, 7%, 8% and 35% in Field2014, Field2015, GH2014 and GH2015, respectively. Little or no difference in predictive ability was observed with sets of 10k, 15k, 20k, 25k, 30k and 36,105 SNPs. Variation in population size did not affect prediction accuracies, so only training population with 352 genotypes (80%) is shown (Fig 2). The effect of marker number and training population size is reported using RR-BLUP because this model maintained prediction accuracies similar to other models and had a lower computational requirement compared with Bayesian models.

**Fig 3:**
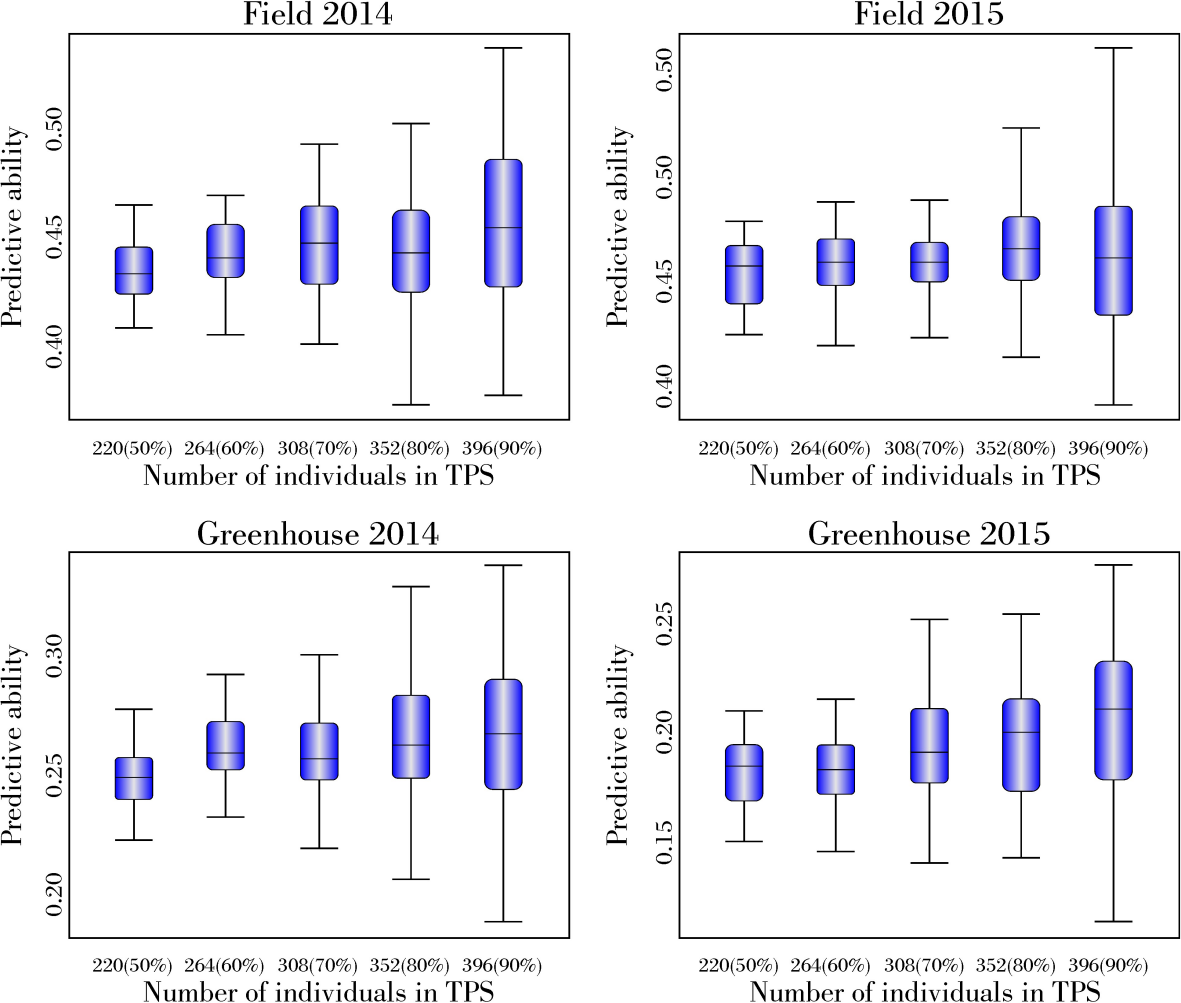
Predictive ability for white mold reaction phenotyped for WM in field and greenhouse screening in 2014 and 2015 using RR-BLUP for differing training population sizes using 5 k SNP markers using 465 diverse soybean accessions.

A slight increase in predictive ability was observed with increased TPS from 220 genotypes (50% of the panel) to 396 genotypes (90% of the panel) (Fig 3). Similar results were obtained for varying sizes of marker sets for all TPSs; therefore, only data for the 5k-marker set are shown (Fig 3).

### Genomic prediction capacity in different experiments

When the same experiment was used to train and validate the model, 10-fold cross-validation was used, and those models were used as control to compare with models performed using different experiments in training and validation population. When one field experiment (Field2014 or Field2015) was used as a TPS and another as the validation population set (VPS), the prediction accuracies were very similar (Table 1). The predictive ability dropped 62% when one greenhouse experiment (GH2014 or GH2015) was used as the TPS and the other as the VPS. When field data were used as the TPS, the predictive ability in the greenhouse decreased. For example, when Field2014 is used as the TPS, the predicted accuracy of GH2014 was 20% less than the predictive ability of GH2014 when GH2014 was the TPS. When greenhouse experiments were used as the TPS, the predictive ability in the field decreased significantly. For example, when GH2014 was used as the TPS, the prediction accuracies for Field2014 and Field2015 were 0.31 and 0.30, respectively, while the best prediction accuracies for Field2014 and Field2015 were 0.44 and 0.47, respectively. The GP model using Field2014 as the TPS accurately predicted the Field2015 phenotype, and vice versa.

**Table 1:** Predictive ability and standard deviation reported using RR-BLUP from white mold evaluation experiments in field and greenhouse (2014 and 2015), when one environment was used to train the model and validated on another environment. The predictive ability (estimated by 10 fold cross-validation) using the same experiment for training and validation population was used as control.

Training\Validation	Field2014	Field2015	GH2014	GH2015
Field2014	0.44 ± 0.05	0.45 ± 0.06	0.20 ± 0.08	0.22 ± 0.08
Field2015	0.46 ± 0.04	0.47 ± 0.07	0.20 ± 0.08	0.20 ± 0.08
GH2014	0.31 ± 0.07	0.30 ± 0.07	0.25 ± 0.07	0.15 ± 0.09
GH2015	0.36 ± 0.06	0.32 ± 0.07	0.17 ± 0.09	0.24 ± 0.08

### Testing GP model

**Fig 4:**
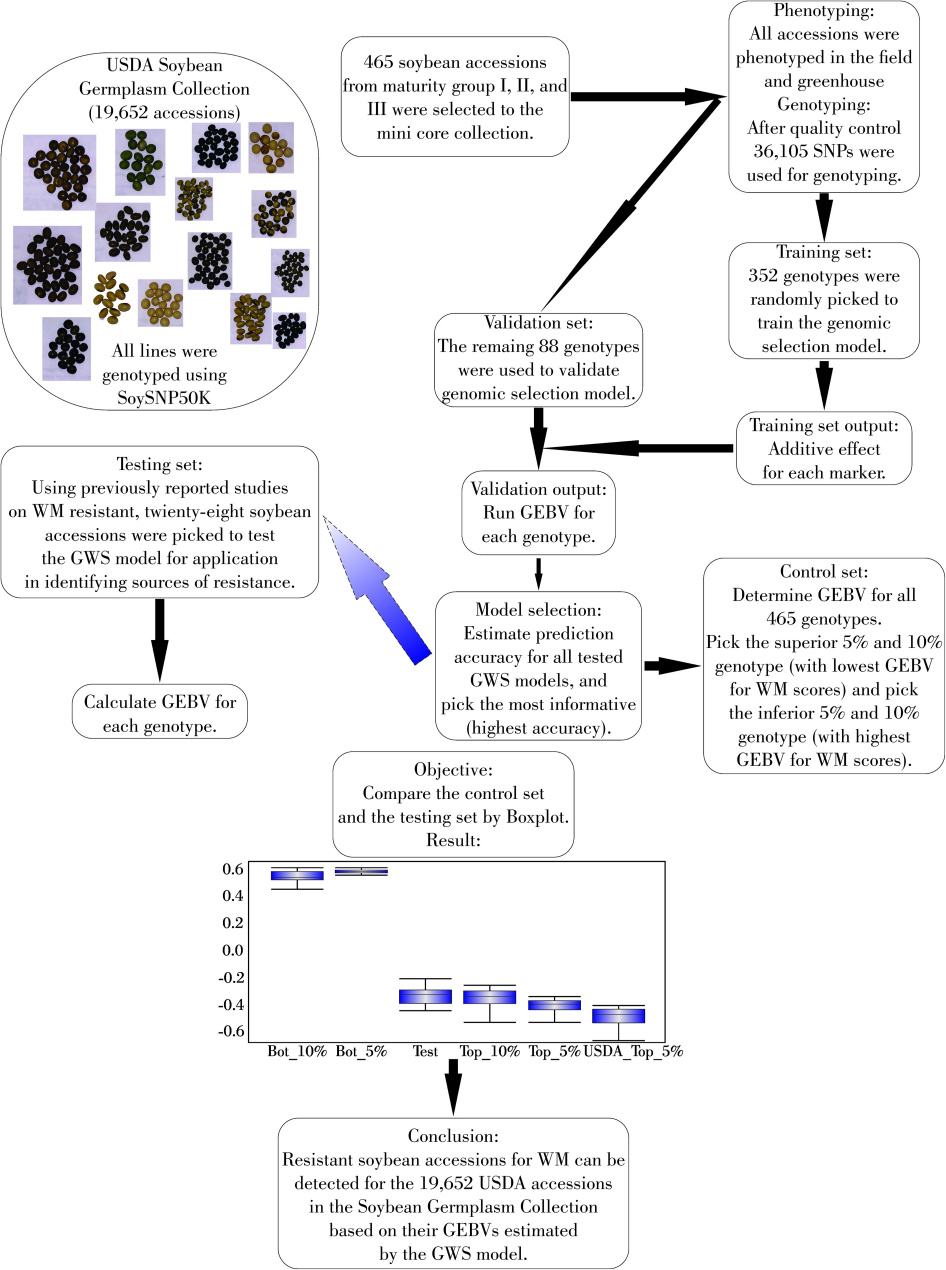
Scheme demonstrating the use of genomic selection models in training, validating, and testing sets. Top– 5% and 10% most resistant accessions for WM found in the present study (F2015); Bottom– 5% and 10% most susceptible accessions for WM found in the present study (F2015); USDA_Top– 5% most resistant accessions found in USDA soybean germplasm collection; Test–Resistant accessions previously reported by other researches. GWS = Genome wide selection, GEBV = Genomic estimated breeding value, WM = white mold.

The GEBV estimated by the GP model using RR-BLUP with 5k markers and 352 genotypes for twenty-nine previously and independently reported resistant accessions was similar to the GEBV of the 10% most resistant genotypes (Fig 4).

The comparison of the 5% most resistant accession in the USDA soybean germplasm collection with the 5% (23 accessions) and 10% (46 accessions) most resistant accessions identified in this study revealed that thirty-five of the fourth-six (76%) accessions were common with the 5% most resistant accessions from the USDA soybean germplasm collection (Fig 4 and S1 Table). The country of origin of the 5% most resistant accessions in the USDA soybean germplasm collection varied: Japan (431 accessions), South Korea (326 accessions) and China (46 accessions) (S5 Fig); and belonged to maturity groups III (114 accessions), IV (241 accessions), V (185 accessions) and VI (124 accessions) (S6 Fig).

## Discussion

### Phenotypic evaluation and GP methods comparison

Variability among accessions for white mold based on phenotypic distribution (Fig 1 and S1 Fig) and PCA (S3 Fig) was identified. The identification and mobilization of useful genetic variation from germplasm bank for use in breeding programs is clearly a necessity not only for sustaining current rates, but also for increasing future rates of crop genetic improvement [33]. And it was also observed that the variability between field and greenhouse experiments were different (Fig 1 and S1 Fig).

Based on the differences between field and greenhouse experiments, they were evaluated for white mold separately and the correlation between them were estimated (S2 Fig). This allowed the comparison of different environments in an attempt to understand the modes of resistance underlying each of them and possibly explain the lack of correlation between field and greenhouse evaluations that has been previously reported [34–39]. These low correlations observed in this study support the fact that greenhouse experiments, although informative, do not correlate as well with field responses. At a correlation coefficient of 0.72, Field2014 and Field2015 observed the highest correlation coefficient between experiments. Therefore, in order to identify new sources of WM resistance, field screenings should be utilized as they are more representative of farmers’ fields.

Moreover, the heritability estimate in this study was similar to previously reported estimates for DSI among recombinant inbred line (RIL) populations which ranged from 0.30 to 0.71 in individual field environments and 0.59 across environments [8]. This heritability means that it is possible to obtain gain with selection in our study.

Since the genetic material used in this study consisted of diverse accessions from the USDA soybean germplasm collection (S3 Fig), it does not represent the pedigreed structure that a soybean breeding program, developing commercial cultivars, will encounter. However, the results from the genetic structure and composition of entries in this study would be applicable to germplasm enhancement programs using diverse collections to obtain parental materials. Therefore, the genetic variability observed in the study panel make it suitable to develop genome wide predictions for the identification of soybean accession resistant to white mold.

There was no difference between GP methods compared in this study (S4 Fig). Several studies have shown that, in comparison with ridge regression methods, more complex statistical methods give little increase in the accuracy of GP for polygenic traits [40–43]. Despite there were no statistics differences between methods in this study, RR-BLUP performed a little better than some Bayesian methods in certain experiments. Moreover the RR-BLUP model has other advantages compared with Bayesian methods such as relative simplicity, reduced computing time, and the well-known optimality properties of mixed models for selection [44].

### Effect of marker density and training population size in the genomic prediction

Models fitted using at least 5K markers were capable enough to predict WM (Fig 2). Poland *et al*. (2012) evaluated the number of markers influencing wheat populations and verified that 1,827 genotyping-by-sequencing (GBS) markers had a similar prediction accuracy to 34,729 GBS markers [45]. Spindel *et al*. (2015) evaluated differently sized SNP subsets from the 73,147 SNP set and verified that there was no significant difference in the best performing GP method for grain yield, days for 50% flowering and plant height [31]. Jarquín *et al*. (2014) evaluated the effect of SNP filtering on accuracy assessed by building a series of G-BLUP models using SNP datasets created by applying combinations of MAF and PMV filtering criteria [17]. Overall, marker filtering criteria did not have a large effect on accuracy for grain yield in soybean, but it had effects on the accuracy for plant height and days to maturity, when was observed that the accuracy improved when more markers were used to train the GP model.

The comparable performance of a limited number of markers (5k) relative to the complete marker data set (36,105) could be due to marker saturation because random markers was selected per chromosome, but the same number of marker were picked in each one, i.e., in the 5k samples, 250 markers were selected randomly per chromosome. With larger linkage disequilibrium (LD), the addition of more markers will not increase the accuracy of predictive models [31]. Therefore, it might be desirable to reduce the SNP numbers to ease computational requirements when predicting individual SNP effects and summing effects for GP. However, more saturated SNP datasets may be more desirable for computing GP of multi-family selection schemes of more diverse germplasm. The RR-BLUP approach is more computationally efficient compared with Bayesian models with computational demands scaling with individual number rather than marker number [17]. Because correlation between the number of markers and predictive ability was not observed in this research, a good GP model to predict WM in soybean can be fitted using about 5k markers in a diverse genotype collection. The use of a small SNP set can lead to cost savings. Use of a uniform or common SNP set will allow consistent utilization of genome wide prediction in the research and breeding programs.

When the training population size increased, a small increase in the predictive ability was observed (Fig 3). Several studies have demonstrated that the accuracy of GP is highly influenced by the population used to calibrate the model [29,32,43,46]. However, Charmet *et al*. [2014] observed that predicted accuracies did not improve significantly with the increase of the training population size when unrelated populations from different breeding programs were merged to create a new population. Similarly, the lack of significant increase in predictive ability with an increased number of individuals in the training population could therefore be explained by the high diversity of soybean lines in this study (S4 Fig).

On the other hand, Boligon *et al*. (2012) concluded that strategies that maximize the phenotypic variance, by selecting individuals from the two-tail distribution, are preferred to those using genotypes with the largest or lowest phenotypic deviation [47]. Thus, capturing most of the phenotypic variance in the training population seems to be the key to increase the prediction ability [32]. Indeed the TPS did not influence the predictive ability because the highest and lowest classes were represented by few individuals (Fig 1). Therefore, it may be useful to choose genotypes that provide equal distribution across the rating scale (resistant, score = 0 to susceptible, score = 3) to compose the training population in future analyses. The variability would then be maximized, the predictive ability improved, and fewer plants should be phenotyped for use in the training population, saving money and time.

### Prediction in different environments

The main objective in GP analysis is to use one generation of data to train the model which would then be used to predict the data of the next generation [40]. Models built using TPS from a field experiment had similar accuracies in predicting results of a different field experiment. The same happened when models were fitted using one greenhouse experiment as the TPS and another as the VPS (Table 1). However, when models fitted using field data as the TPS were used to predict greenhouse performance, the predictive ability was low, and vice versa.

Field and greenhouse experiments differed for inoculation and phenotypic evaluation. Field2014 and Field2015 experiments were inoculated when plots that had reached crop growth stage R1, while GH2014 and GH2015 were inoculated when the third trifoliate was fully expanded, approximately four and six weeks after transplanting respectively. Moreover Field2014 and Field2015 was rated when plants reached the growth stage R5, about 30 days after inoculation (DAI), while GH2014 and GH2015 were rated 14 DAI. Additionally, genetic mechanism of WM resistance is likely not common between the field and greenhouse screening tests. These differences may explain why prediction ability in field was twice than greenhouse.

The models derived from field data had prediction accuracies twice that of those derived from greenhouse data (Table 1 and S4 Fig). This may be explained by the different number of plants per genotype used in each experiment. In the greenhouse experiment, one or two plants per replication were phenotyped in 2015 and 2014, respectively, while the number of plants phenotyped in the field was higher. The lower number of plants in the greenhouse experiments perhaps increase the experiment error, which resulted in the lower predictive ability of predictive models using greenhouse data.

Good predictive ability was observed when the same type of environment was used. The phenotypic correlation between environments ranged from 0.15 (GH2014 and GH2015) to 0.46 (Field2014 and Field2015) (Table 1). Lopez-Cruz *et al*. (2015) compared models with and without the G x E interaction to estimate prediction accuracy for yield in wheat [48]. They concluded that the across-environment model (the same method used in this study) was the worst method to predict between different environments when the correlation between two environments was less than 0.4. Their findings supports our observation and highlights the importance of considering G x E when analyzing multi-environmental data and involving G x E interactions in the model for increasing the predictive ability [48].

### Testing GP model

Twenty-nine previously reported resistant PI lines were compared with our results. Those resistant PI lines were: PI391589B, PI507352, PI561345, PI196157, PI398637, PI358318A, PI189919, PI189861, PI437527, PI549066, PI567157A, PI416776, PI561331, PI437764, PI507353, PI548312, PI504502, PI437072, PI194634, PI281850, PI423941, PI423949, PI424242, PI458520, PI503336, PI593972, PI593973, PI603148 and PI243547 [49]. However, we observed that just four PI lines (PI424242, PI398637, PI281850 and PI549066) previously reported were in the 5% most resistant accessions from the entire USDA soybean germplasm collection, while 23 PI lines were in the 10% most resistant accessions. Several factors can influence these results since PI lines previously identified as resistant were reported on phenotypic values, and may be influenced by environment conditions, pathogen infection, inoculation procedure, or evaluation methods. Therefore, based on GWS outcomes, there are several genotypes in the USDA soybean germplasm collection that are predicted to be more resistant than the previously identified and reported WM resistant PI accessions, and these PI lines need to tested for obtain validation. GP model based on the training population and marker density analyses (RR-BLUP using 5k SNPs– 250 SNPs per chromosome and 352 genotypes in the TPS) in the study accurately predicted WM resistance in PI lines in the USDA soybean germplasm collection that had already been previously reported to be resistant (Fig 4). This suggests that GP analysis using diverse accessions from the germplasm–genebank collection for different crop species and traits needs to be explored to identify useful accessions as parental sources in breeding programs.

Most resistant accessions from the USDA soybean germplasm collection have different countries of origin (S5 Fig) and belonged to different maturities (S6 Fig). Although, our GP model was developed using Maturity groups I, II and III [50], soybean accessions were accurately predicted and validated to be resistant in different maturity groups. These results suggest that the prediction ability is not dependent on maturity group, and this approach needs to be tested in other crop species and biotic stresses, and also would be a useful strategy for gene bank collections to identify selective accessions for further phenotypic validation for use as parents in genetic enhancement programs.

Therefore, we concluded that for WM GP, results from the different GP models did not vary significantly, and minimal response was observed for changes in TPS and marker numbers. GP approaches were shown to be useful to the breeder to assist in selecting among the USDA soybean germplasm collection. The results from this research will assist the establishment of GP approaches for germplasm enhancement using plant introductions and gene back accessions to help maximize the useful genetic diversity for disease resistance breeding.

## Material and methods

### Genetic materials

This study included 465 PIs from the USDA soybean germplasm collection and consisted of 144, 168, and 153 accessions of maturity groups (MG) I, II, and III, respectively. This panel was a subgroup of a large *Glycine max* core collection of 1,685 soybean genotypes that represented the genetic diversity of the entire USDA soybean germplasm collection as determined through pedigree and marker allele analysis [51]. Accessions originated from twenty-seven different countries/regions: China, Japan, Russia, South Korea, North Korea, France, Taiwan, Georgia, Belgium, Algeria, Austria, Eastern Europe, Germany, Hungary, Indonesia, Iran, Moldova, Morocco, Poland, Portugal, Romania, Turkey, United States, Ukraine, Uzbekistan, Vietnam, and Yugoslavia. Twenty-eight accessions were of unknown origin.

### Phenotyping for white mold resistance

#### Field

Each soybean accession was planted in a one-row plot, 1.50 m long arranged in a randomized complete block design with two replications with 15 seeds/m and 24 seeds/m in 2014 and 2015 respectively. Planting was done with a customized ALMACO Cone Plot Planter on 23 May 2014 (experiment referred as Field2014) and 01 June 2015 (experiment referred as Field2015). WM inoculum preparation, inoculation, and rating were modified from the cotton pad method described by Bastien *et al*. (2014). *S*. *sclerotiorum* cultures were started from surface-sterilized sclerotia obtained from a field in Northeast Iowa in 2013 [52]. Inoculation was done in each row at the R1 growth stage [53] by placing a mycelium-soaked cotton ball on the lowest flower-bearing petiole of each plant. Fields were irrigated by overhead sprinklers until the ratings were completed in order to create an epiphytotic disease nursery. When plants had reached the R5 growth stage [29–33 days after inoculation (DAI)], WM disease ratings were taken according to the methodology described in a previous study [54]. The disease severity scale for each plant was: 0 = no symptoms, 1 = lateral branches showing lesions, 2 = lesions on the main stem, and 3 = lesions on the main stem resulting in poor podfill or plant death. The disease severity was used to calculate the disease severity index (DSI) of each plot. The DSI ranged from 0, no disease, to 100, all plants rated were dead or had poor pod-fill due to disease.

$$DSI=\frac{\sum \left(rating\;of\;each\;plant\right)}{3*(number\;of\;plants\;rated)}*100$$ (1)

No specific permissions were required for field locations/activities, as experiment sites are managed by the Iowa State University and university researchers are permitted to perform experiments as described in this study. Field studies did not involve endangered or protected species.

#### Greenhouse

In 2014, the greenhouse experiment was planted using a randomized complete block design with two replications (GH2014). An experimental unit consisted of one plant. Plants were inoculated at the V3 growth stage using a cut-petiole method, which is preferred for indoor screening [55]. In 2015 (GH2015), inoculation was done at the V5 growth stage, and the experiment consisted of one replication with two plants per experimental unit. WM infested plants in both years were rated for 3 days after inoculation (DAI). Disease severity was based on the scale previously described. In 2015, the second replication was lost due to greenhouse malfunction. Since greenhouse is a controlled environment, fewer replications are sufficient and therefore we retained 2015 data. Greenhouse experiments were conducted to complement field studies, as the greenhouse protocol allows for earlier assessment of disease and is less time and resource intensive.

### Genotypic data

All 465 plant accessions had been previously genotyped on an Illumina Infinium SoySNP50K BeadChip [56], and the data is publically accessible at SoyBase (http://soybase.org). SNPs with a missing rate greater than 10% were excluded from further analyses and the remaining missing data were imputed using BEAGLE version 3.3.1 with default parameter settings [57,58]. The remaining missing data were imputed, and SNPs with a minor allele frequency (MAF) lower than 0.05 were ruled out, and these quality control steps left 36,105 SNPs.

### Statistics analysis

A completely random genotype x environment model were used to calculate variance components of individual factors ($\sigma_{G}^{2}$, $\sigma_{GE}^{2}$, and $\sigma_{e}^{2}$) is described below:

$$y_{ij}=\mu +G_{i}+E_{j}+{GE}_{ij}+\epsilon_{ijk}$$ (2)

In which *y*_*ij*_ is the phenotypic value for the i^th^ genotype in the j^th^ environment; *μ* is the overall mean; *G*_*i*_ is the random effect of the i^th^ genotype; *E*_*j*_ is the random effect of the j^th^ environment; *GE*_*ij*_ is the GenotypexEnvironment effect assigned as random; and *ϵ*_*ijk*_ is the residual error.

Variance components were calculated using the *lmer* function in R package lme4 [59]. Broad-sense heritability (*H*) was calculated across environments for DSI in field environments. The equation used was:

$$H=\frac{\sigma_{G}^{2}}{\sigma_{G}^{2}+\frac{\sigma_{GE}^{2}}{e}+\frac{\sigma_{e}^{2}}{re}}$$ (3)

Where *G* is genotype and *E* is environment, *e* is the number of environments and *r* is the number of replicates.

All experiments and years were analyzed separately. When an experiment was replicated and the disease response was ordinal, a logistic mixed model analysis was used to obtain BLUP for each accession. A mixed model approach was chosen to accommodate the unbalanced sample size among cultivars due to emergence rates. The analysis was performed using the *clmm* function (part of the ordinal package; [59]) executed in the R statistical analysis software [60]. The model for both field experiments (Field2014 and Field2015) was

$$Y_{jklm}∼Multinomial(1,\pi_{ijklm})$$ (4)

$$logit(\pi_{ijklm})=\theta_{i}+R_{j}+A_{k}+RA_{jk}+D_{l}+S_{m}+DS_{lm}$$ (5)

where *θ*_*i*_ is the intercept for the *i*th response category (*i* = 0,1,2), *R*_*j*_ is the effect of the *j*th replication, *A*_*k*_ is the effect of the *k*th accession, *RA*_*jk*_ is replication × accession interaction, *D*_*l*_ is the effect of the *l*th inoculation date (inoculation was done per plot at the R1 growth stage therefore inoculations were made on multiple dates in the entire nursery), *S*_*m*_ is the effect of the *m*th row, and *DS*_*lm*_ is inoculation date × row interaction. The term *Y*_*jklm*_ represents a vector of ratings and *π*_*ijklm*_ is the probability that *Y*_*jklm*_ will be rated at or below the *i*th response category. Replication was assumed to be a fixed effect, and all other terms were assumed to be random effects.

Similarly, the model for GH2014 and GH2015 was

$$Y_{jk}∼Multinomial(1,\pi_{ijk})$$ (6)

$$logit(\pi_{ijk})=\theta_{i}+R_{j}+A_{k}$$ (7)

Due to lack of replication in GH2015 experiments, phenotypic data for each accession consisted of the average response of the two plants.

Principal components analysis (PCA) was performed to estimate the diversity among soybean accessions. PCA was calculated based on all SNPs. MANOVA was used to estimate correlations between experiments and years based on GEBV estimated by GP models. PCA and MANOVA were performed using the stats package in R software.

### Genomic prediction models

Six GP methods were used to analyze greenhouse and field experiments: ridge regression best linear unbiased prediction (RR-BLUP), Bayes A, Bayes B, Bayes Cπ, Bayesian LASSO (BLASSO), and Reproducing Kernel Hilbert Spaces (RKHS) regression. The genetic value estimated by the logistic regression was used as input in all GP models.

RR-BLUP, Bayes A, and Bayes B were described by in a previous study [11]. RR-BLUP assumes that each marker has an equal variance, V_G_/M, where V_G_ is the genetic variance, and M is the number of markers. In Bayes A, each marker effect is drawn from a normal distribution with its own variance: N (0, $\sigma_{gi}^{2}$). The variance parameters are in turn sampled from a scaled inverse chi-squared distribution. In the Bayes B approach, the prior for the proportion of markers associated with zero phenotypic variance, π, is assumed unknown. Other prior hyperparameters for marker variance components in Bayes A and Bayes B were as given in Meuwissen *et al*. (2001)[11].

Bayes Cπ assumes common marker variances and allows for some markers to have no effect [61]. Additionally, Bayes Cπ jointly estimates π from the training data to avoid an incorrect π that can negatively affect prediction accuracy [62].

In BLASSO [63], marker effects are assigned independent Gaussian priors with marker-specific variances ($\sigma_{e}^{2}\tau_{j}^{2}$). At the next level of the hierarchical model, the $\tau_{j}^{2}$s are assigned exponential priors $EXP[\tau_{j}^{2}|\lambda^{2}].$ At a deeper level of the hierarchy, *λ*^2^ is assigned a gamma prior with rate (δ) and shape (r) which, in this study, was the default in the Bayesian Generalized Linear Regression (BGLR) package in R software. Finally, inverse chi-square priors were assigned to the variance parameters, and the scale and degree of freedom parameters were set to S_u_ = S_e_ = 1 and d:f_:e_ = d:f_:u_ = 4, respectively.

In RKHS regression, genetic values are viewed as a Gaussian process. When markers and a pedigree are available, genetic values are modeled as the sum of two components: *g*_*i*_ *= u*_*i*_ *+ f*_*i*,_ where *u*_*i*_ is the mean, and *f*_*i*_ is a Gaussian process with a (co)variance function proportional to the evaluations of a reproducing kernel, K(*x*_*i*_, *x*_*j*_), evaluated in marker genotypes. Here, *x*_*i*_ and *x*_*j*_ are vectors of marker genotype codes for the i^th^ and j^th^ individuals, respectively. All hyperparameters were assumed following [64]. The genomic relationship matrix was estimated using the function A.mat (rrBLUP package) in R software.

### Cross validation

The ten-fold cross-validation (CV) was performed to avoid an inflated estimate of the predictive ability of GP. In ten-fold CV, the genotypes set was divided into ten equally-sized subgroups. Of these, nine subgroups were used as the training population to fit each prediction model while the remaining subgroup was used as the validation population to assess the correlation between the observed and predicted trait values. This process was repeated ten times, with each subgroup being the validation population exactly once. This process was repeated fifty times by randomizing the genotypes and re-forming the folds. The mean of the 50 correlation coefficients was reported.

Predictive ability was assessed using the Pearson correlation of the predicted GEBV and the genetic value estimated by the logistic regression in the validation population [65].

### Marker density and training population size

The effect of marker numbers on GP predictive ability was determined through ten-fold cross-validation by including random samples of 100, 200, 500, 1k, 5k, 10k, 15k, 20k, 25k, and 30k SNPs from the full marker set. These samples were compared with the model which used 36,105 markers. For this analysis, the marker file was divided in 20 sets, where each set was composed with all SNPs in each chromosome. Then the same number of SNPs was picked in each set, i.e., each chromosome. The SNPs were picked randomly within each set. All codes were run in R software.

The impact of TPS on predictive ability was evaluated using training sets of variable sizes (220 genotypes [50% of the population], 264[60% of the population], 308[70% of the population], 352[80% of the population], and 396[90% of the population] genotypes). All marker subsets described above were used to evaluate these training population sizes. The predictive ability was estimated for each combination of marker number and TPS.

### Genomic prediction capacity in different experiment

To verify the prediction capacity of the GP model to predict WM in unrelated experiments, each experiment (two field and GH experiments each) was used as the training population to validate the other three experiments. The RR-BLUP method was fitted using all markers, and 352 (80% of total population) genotypes were used in the training population.

In order to test the ability of the GP model to identify WM resistant genotypes from the USDA soybean germplasm collection, twenty-nine plant-introduction (PI) soybean accessions, previously reported to have WM resistance [49], were chosen. Marker data were available on these twenty-nine soybean PI accessions from the same SNPs run on the 465 PI accessions used in the model. Using the GP model and available marker information, the GEBV was obtained on these twenty-nine accessions. Boxplot was used to compare twenty-nine plant-introduction (PI) soybean accessions with the 5% (23 accessions) and 10% (46 accessions) most resistant PI lines and the 5% (23 accessions) and 10% (46 accessions) most susceptible PI lines found in our results. The 5% and 10% more resistant PI lines was assigned being the accessions with the lowest GEBV, meanwhile the 5% and 10% more susceptible PI lines was assigned being the accessions with the highest GEBV based on the GP model.

SNPs effect estimated in the training population by RR-BLUP using the average between Field2014 and Field2015 was used to predict the entire USDA soybean germplasm collection (19,652 accessions). The average between Field2014 and Field2015 was chosen to perform this analysis because the field experiment provided the greatest precision based on the estimated broad-sense heritability and GWS accuracy. The 5% (982 accessions) most resistant accessions were selected and compared with the 5% and 10% most resistant accessions selected in our study.

### Software and computer information

All analyses were executed in R software [60]. RR-BLUP was performed using the rrBLUP package [66]. BayesA, BayesB, BayesCπ, and RKHS were performed using the BGLR package [67]. BLASSO was performed using the BLR (Bayesian Linear Regression) package [68].

A total of 20,000 burn-ins (number of iterations before the Bayesian analysis convergence) and 40,000 saved iterations, obtained from the Markov chain Monte Carlo (MCMC) method, was used in all Bayesian methods. The convergence of Bayesian models was checked by inspecting trace plots of variance parameters.

## Supporting information

S1 Fig White mold (caused by *Sclerotinia sclerotiorum* (Lib.) de Bary) phenotypic data distribution of 465 soybean accessions tested in field and greenhouse specialized tests. (TIF)

S2 Fig Genotypic correlation based on the genomic estimated breeding value (GEBV) between all experiments from 465 soybean accessions evaluated for reaction to white mold (caused by *Sclerotinia sclerotiorum* (Lib.) de Bary) in field and greenhouse (2014 and 2015) nurseries. (TIF)

S3 Fig Principal component analysis showing of the first two components based on 36,105 SNPs run on the 465 accessions in the soybean mini-core panel. (TIF)

S4 Fig Predictive ability of the GP methods for 465 soybean diverse accessions phenotyped for white mold (caused by *Sclerotinia sclerotiorum* (Lib.) de Bary) and genotyped using 36,105 SNP markers and a training set composed of 352 genotypes. The range in predictive ability is among the 50 replicates of the cross validation experiment
BA–Bayes A; BB–Bayes B; BC–Bayes Cπ; BL–Bayesian LASSO; GB–Genomic Best Linear Unbiased Prediction; RK–Reproducing Kernel Hilbert Space Regression; BR–Best Linear Unbiased Prediction.
(TIF)

S5 Fig Country of origin of the 5% most resistant accessions for white mold (caused by *Sclerotinia sclerotiorum* (Lib.) de Bary) from the entire USDA soybean germplasm collection. (TIF)

S6 Fig Maturity group of the 5% most resistant accessions for white mold (caused by *Sclerotinia sclerotiorum* (Lib.) de Bary) from the entire USDA soybean germplasm collection. (TIF)

S1 Table 5% most resistant soybean accessions for the entire USDA soybean germplasm collection and their GEBVs. (DOCX)

## References

[1] Boland GJ, Hall R (1994) Index of Plant Hosts of Sclerotinia-Sclerotiorum. Canadian Journal of Plant Pathology-Revue Canadienne De Phytopathologie 16: 93–108.

[2] Koch L, Hildebrand A (1944) Soybean diseases in southwestern Ontario in 1943. Soybean diseases in southwestern Ontario in 1943: 29–32.

[3] Koenning SR, Wrather JA (2010) Suppression of soybean yield potential in the continental United States by plant diseases from 2006 to 2009. Plant Health Progress 10.

[4] Fernando WGD, Ramarathnam R, Krishnamoorthy AS, Savchuk SC (2005) Identification and use of potential bacterial organic antifungal volatiles in biocontrol. Soil Biology & Biochemistry 37: 955–964.

[5] Navi SS, Yang X-B, Pecinovsky KT (2009) Efficacy Results of Fungicides on Soybean White Mold Control.

[6] Zhao X, Han YP, Li YH, Liu DY, Sun MM, et al (2015) Loci and candidate gene identification for resistance to Sclerotinia sclerotiorum in soybean (Glycine max L. Merr.) via association and linkage maps. Plant Journal 82: 245–255. doi: 10.1111/tpj.12810 25736370 10.1111/tpj.12810

[7] Peltier AJ, Bradley CA, Chilvers MI, Malvick DK, Mueller DS, et al (2012) Biology, yield loss and control of Sclerotinia stem rot of soybean. Journal of Integrated Pest Management 3: B1–B7.

[8] Kim HS, Diers BW (2000) Inheritance of partial resistance to Sclerotinia stem rot in soybean. Crop Science 40: 55–61.

[9] Collard BCY, Mackill DJ (2008) Marker-assisted selection: an approach for precision plant breeding in the twenty-first century. Philosophical Transactions of the Royal Society B-Biological Sciences 363: 557–572. 10.1098/rstb.2007.2170 PMC2610170 17715053

[10] Mudge J, Cregan PB, Kenworthy JP, Kenworthy WJ, Orf JH, et al (1997) Two microsatellite markers that flank the major soybean cyst nematode resistance locus. Crop Science 37: 1611–1615.

[11] Meuwissen THE, Hayes BJ, Goddard ME (2001) Prediction of total genetic value using genome-wide dense marker maps. Genetics 157: 1819–1829. 11290733 10.1093/genetics/157.4.1819 PMC1461589

[12] Lorenz AJ, Chao SM, Asoro FG, Heffner EL, Hayashi T, et al (2011) Genomic Selection in Plant Breeding: Knowledge and Prospects. Advances in Agronomy, Vol 110 110: 77–123.

[13] Burgueno J, de los Campos G, Weigel K, Crossa J (2012) Genomic Prediction of Breeding Values when Modeling Genotype x Environment Interaction using Pedigree and Dense Molecular Markers. Crop Science 52: 707–719.

[14] Heslot N, Yang HP, Sorrells ME, Jannink JL (2012) Genomic Selection in Plant Breeding: A Comparison of Models. Crop Science 52: 146–160.

[15] Lorenz AJ, Smith KP, Jannink JL (2012) Potential and Optimization of Genomic Selection for Fusarium Head Blight Resistance in Six-Row Barley. Crop Science 52: 1609–1621.

[16] Heffner EL, Jannink JL, Iwata H, Souza E, Sorrells ME (2011) Genomic Selection Accuracy for Grain Quality Traits in Biparental Wheat Populations. Crop Science 51: 2597–2606.

[17] Jarquin D, Kocak K, Posadas L, Hyma K, Jedlicka J, et al (2014) Genotyping by sequencing for genomic prediction in a soybean breeding population. Bmc Genomics 15. 10.1186/1471-2164-15-740 PMC4176594 25174348

[18] Bao Y, Vuong T, Meinhardt C, Tiffin P, Denny R, et al (2014) Potential of Association Mapping and Genomic Selection to Explore PI 88788 Derived Soybean Cyst Nematode Resistance. Plant Genome 7.

[19] Shu YJ, Yu DS, Wang D, Bai X, Zhu YM, et al (2013) Genomic selection of seed weight based on low-density SCAR markers in soybean. Genetics and Molecular Research 12: 2178–2188. doi: 10.4238/2013.July.3.2 23884761 10.4238/2013.July.3.2

[20] Zhang JP, Song QJ, Cregan PB, Jiang GL (2016) Genome-wide association study, genomic prediction and marker-assisted selection for seed weight in soybean (Glycine max). Theoretical and Applied Genetics 129: 117–130. doi: 10.1007/s00122-015-2614-x 26518570 10.1007/s00122-015-2614-x PMC4703630

[21] Bao Y, Kurle JE, Anderson G, Young ND (2015) Association mapping and genomic prediction for resistance to sudden death syndrome in early maturing soybean germplasm. Molecular Breeding 35. 10.1007/s11032-015-0324-3 PMC4434860 25999779

[22] Goddard ME, Hayes BJ (2007) Genomic selection. Journal of Animal Breeding and Genetics 124: 323–330. doi: 10.1111/j.1439-0388.2007.00702.x 18076469 10.1111/j.1439-0388.2007.00702.x

[23] Daetwyler HD, Villanueva B, Woolliams JA (2008) Accuracy of Predicting the Genetic Risk of Disease Using a Genome-Wide Approach. Plos One 3. 10.1371/journal.pone.0003395 PMC2561058 18852893

[24] Daetwyler HD, Pong-Wong R, Villanueva B, Woolliams JA (2010) The Impact of Genetic Architecture on Genome-Wide Evaluation Methods. Genetics 185: 1021–1031. doi: 10.1534/genetics.110.116855 20407128 10.1534/genetics.110.116855 PMC2907189

[25] Lorenzana RE, Bernardo R (2009) Accuracy of genotypic value predictions for marker-based selection in biparental plant populations. Theoretical and Applied Genetics 120: 151–161. doi: 10.1007/s00122-009-1166-3 19841887 10.1007/s00122-009-1166-3

[26] Grattapaglia D, Resende MDV (2011) Genomic selection in forest tree breeding. Tree Genetics & Genomes 7: 241–255.

[27] Guo ZG, Tucker DM, Lu JW, Kishore V, Gay G (2012) Evaluation of genome-wide selection efficiency in maize nested association mapping populations. Theoretical and Applied Genetics 124: 261–275. doi: 10.1007/s00122-011-1702-9 21938474 10.1007/s00122-011-1702-9

[28] Heffner EL, Jannink JL, Sorrells ME (2011) Genomic Selection Accuracy using Multifamily Prediction Models in a Wheat Breeding Program. Plant Genome 4: 65–75.

[29] Albrecht T, Wimmer V, Auinger HJ, Erbe M, Knaak C, et al (2011) Genome-based prediction of testcross values in maize. Theoretical and Applied Genetics 123: 339–350. doi: 10.1007/s00122-011-1587-7 21505832 10.1007/s00122-011-1587-7

[30] Rabier CE, Barre P, Asp T, Charmet G, Mangin B (2016) On the Accuracy of Genomic Selection. Plos One 11. 10.1371/journal.pone.0156086 PMC4913905 27322178

[31] Spindel J, Begum H, Akdemir D, Virk P, Collard B, et al (2015) Genomic Selection and Association Mapping in Rice (Oryza sativa): Effect of Trait Genetic Architecture, Training Population Composition, Marker Number and Statistical Model on Accuracy of Rice Genomic Selection in Elite, Tropical Rice Breeding Lines. Plos Genetics 11. 10.1371/journal.pgen.1004982 PMC4334555 25689273

[32] Isidro J, Jannink JL, Akdemir D, Poland J, Heslot N, et al (2015) Training set optimization under population structure in genomic selection. Theoretical and Applied Genetics 128: 145–158. doi: 10.1007/s00122-014-2418-4 25367380 10.1007/s00122-014-2418-4 PMC4282691

[33] Sehgal D, Vikram P, Sansaloni CP, Ortiz C, St Pierre C, et al (2015) Exploring and Mobilizing the Gene Bank Biodiversity for Wheat Improvement. Plos One 10. 10.1371/journal.pone.0132112 PMC4503568 26176697

[34] Boland GJ, Hall R (1986) Growthroom Evaluation of Soybean Cultivars for Resistance to Sclerotinia-Sclerotiorum. Canadian Journal of Plant Science 66: 559–564.

[35] Chun D, Kao LB, Lockwood JL, Isleib TG (1987) Laboratory and Field Assessment of Resistance in Soybean to Stem Rot Caused by Sclerotinia-Sclerotiorum. Plant Disease 71: 811–815.

[36] Kim HS, Sneller CH, Diers BW (1999) Evaluation of soybean cultivars for resistance to Sclerotinia stem rot in field environments. Crop Science 39: 64–68.

[37] Hoffman DD, Diers BW, Hartman GL, Nickell CD, Nelson RL, et al (2002) Selected soybean plant introductions with partial resistance to Sclerotinia sclerotiorum. Plant Disease 86: 971–980. 10.1094/PDIS.2002.86.9.971 30818558

[38] Nelson BD, Helms TC, Olson MA (1991) Comparison of Laboratory and Field Evaluations of Evaluations of Resistance in Soybean to Sclerotinia-Sclerotiorum. Plant Disease 75: 662–665.

[39] Wegulo SN, Yang XB, Martinson CA (1998) Soybean cultivar responses to Sclerotinia sclerotiorum in field and controlled environment studies. Plant Disease 82: 1264–1270. 10.1094/PDIS.1998.82.11.1264 30845417

[40] Bernardo R, Yu JM (2007) Prospects for genomewide selection for quantitative traits in maize. Crop Science 47: 1082–1090.

[41] Daetwyler HD, Calus MPL, Pong-Wong R, de los Campos G, Hickey JM (2013) Genomic Prediction in Animals and Plants: Simulation of Data, Validation, Reporting, and Benchmarking. Genetics 193: 347-+. doi: 10.1534/genetics.112.147983 23222650 10.1534/genetics.112.147983 PMC3567728

[42] Cleveland MA, Hickey JM, Forni S (2012) A Common Dataset for Genomic Analysis of Livestock Populations. G3-Genes Genomes Genetics 2: 429–435. 10.1534/g3.111.001453 PMC3337471 22540034

[43] Clark SA, Hickey JM, Daetwyler HD, van der Werf JHJ (2012) The importance of information on relatives for the prediction of genomic breeding values and the implications for the makeup of reference data sets in livestock breeding schemes. Genetics Selection Evolution 44. 10.1186/1297-9686-44-4 PMC3299588 22321529

[44] Fernando RL, Gianola D (1986) Optimal Properties of the Conditional Mean as a Selection Criterion. Theoretical and Applied Genetics 72: 822–825. doi: 10.1007/BF00266552 24248207 10.1007/BF00266552

[45] Poland J, Endelman J, Dawson J, Rutkoski J, Wu SY, et al (2012) Genomic Selection in Wheat Breeding using Genotyping-by-Sequencing. Plant Genome 5: 103–113.

[46] Pszczola M, Strabel T, Mulder HA, Calus MPL (2012) Reliability of direct genomic values for animals with different relationships within and to the reference population. Journal of Dairy Science 95: 389–400. doi: 10.3168/jds.2011-4338 22192218 10.3168/jds.2011-4338

[47] Boligon AA, Long N, Albuquerque LG, Weigel KA, Gianola D, et al (2012) Comparison of selective genotyping strategies for prediction of breeding values in a population undergoing selection. Journal of Animal Science 90: 4716–4722. doi: 10.2527/jas.2012-4857 23372045 10.2527/jas.2012-4857

[48] Lopez-Cruz M, Crossa J, Bonnett D, Dreisigacker S, Poland J, et al (2015) Increased Prediction Accuracy in Wheat Breeding Trials Using a Marker x Environment Interaction Genomic Selection Model. G3-Genes Genomes Genetics 5: 569–582. 10.1534/g3.114.016097 PMC4390573 25660166

[49] Iquira E, Humira S, Francois B (2015) Association mapping of QTLs for sclerotinia stem rot resistance in a collection of soybean plant introductions using a genotyping by sequencing (GBS) approach. Bmc Plant Biology 15. 10.1186/s12870-014-0408-y PMC4304118 25595526

[50] Greilhuber J, Obermayer R (1997) Genome size and maturity group in Glycine max (soybean). Heredity 78: 547–551.

[51] Oliveira MF, Nelson RL, Geraldi IO, Cruz CD, de Toledo JFF (2010) Establishing a soybean germplasm core collection. Field Crops Research 119: 277–289.

[52] Bastien M, Sonah H, Belzile F (2014) Genome Wide Association Mapping of Sclerotinia sclerotiorum Resistance in Soybean with a Genotyping-by-Sequencing Approach. Plant Genome 7. 10.1186/s12870-020-02401-8 PMC7333386 32380949

[53] Pedersen P, Kumudini S, Board J, Conley S (2004) Soybean growth and development: Iowa State University, University Extension Ames, IA.

[54] Grau CR, Radke VL, Gillespie FL (1982) Resistance of Soybean Cultivars to Sclerotinia-Sclerotiorum. Plant Disease 66: 506–508.

[55] Guo B, Wang Y, Sun X, Tang K (2008) Bioactive natural products from endophytes: A review. Applied Biochemistry and Microbiology 44: 136–142. 18669256

[56] Song QJ, Hyten DL, Jia GF, Quigley CV, Fickus EW, et al (2015) Fingerprinting Soybean Germplasm and Its Utility in Genomic Research. G3-Genes Genomes Genetics 5: 1999–2006. 10.1534/g3.115.019000 PMC4592982 26224783

[57] Browning BL, Browning SR (2007) Efficient multilocus association testing for whole genome association studies using localized haplotype clustering. Genetic Epidemiology 31: 365–375. doi: 10.1002/gepi.20216 17326099 10.1002/gepi.20216

[58] Browning BL, Browning SR (2009) A Unified Approach to Genotype Imputation and Haplotype-Phase Inference for Large Data Sets of Trios and Unrelated Individuals. American Journal of Human Genetics 84: 210–223. doi: 10.1016/j.ajhg.2009.01.005 19200528 10.1016/j.ajhg.2009.01.005 PMC2668004

[59] Christensen RHB (2010) ordinal—regression models for ordinal data. R package version 22.

[60] Team RC (2012) R: A language and environment for statistical computing.

[61] Jannink JL, Lorenz AJ, Iwata H (2010) Genomic selection in plant breeding: from theory to practice. Briefings in Functional Genomics 9: 166–177. doi: 10.1093/bfgp/elq001 20156985 10.1093/bfgp/elq001

[62] Gianola D, de los Campos G, Hill WG, Manfredi E, Fernando R (2009) Additive Genetic Variability and the Bayesian Alphabet. Genetics 183: 347–363. doi: 10.1534/genetics.109.103952 19620397 10.1534/genetics.109.103952 PMC2746159

[63] de los Campos G, Naya H, Gianola D, Crossa J, Legarra A, et al (2009) Predicting Quantitative Traits With Regression Models for Dense Molecular Markers and Pedigree. Genetics 182: 375–385. doi: 10.1534/genetics.109.101501 19293140 10.1534/genetics.109.101501 PMC2674834

[64] de los Campos G, Gianola D, Rosa GJM, Weigel KA, Crossa J (2010) Semi-parametric genomic-enabled prediction of genetic values using reproducing kernel Hilbert spaces methods. Genetics Research 92: 295–308. doi: 10.1017/S0016672310000285 20943010 10.1017/S0016672310000285

[65] Riedelsheimer C, Czedik-Eysenberg A, Grieder C, Lisec J, Technow F, et al (2012) Genomic and metabolic prediction of complex heterotic traits in hybrid maize. Nature Genetics 44: 217–220. doi: 10.1038/ng.1033 22246502 10.1038/ng.1033

[66] Endelman JB (2011) Ridge Regression and Other Kernels for Genomic Selection with R Package rrBLUP. Plant Genome 4: 250–255.

[67] Pérez P, de los Campos G (2013) BGLR: a statistical package for whole genome regression and prediction. R package version 1. 10.1534/genetics.114.164442 PMC4196607 25009151

[68] Pérez P, de los Campos G, Crossa J, Gianola D (2010) Genomic-enabled prediction based on molecular markers and pedigree using the Bayesian linear regression package in R. The plant genome 3: 106–116. doi: 10.3835/plantgenome2010.04.0005 21566722 10.3835/plantgenome2010.04.0005 PMC3091623

