# Supplementary figures and images for: Leveraging genomic prediction to scan germplasm collection for crop improvement
Source: PLoS One. 2017 Jun 9;12(6):e0179191. doi: 10.1371/journal.pone.0179191 (PMC5466325; doi:10.1371/journal.pone.0179191)

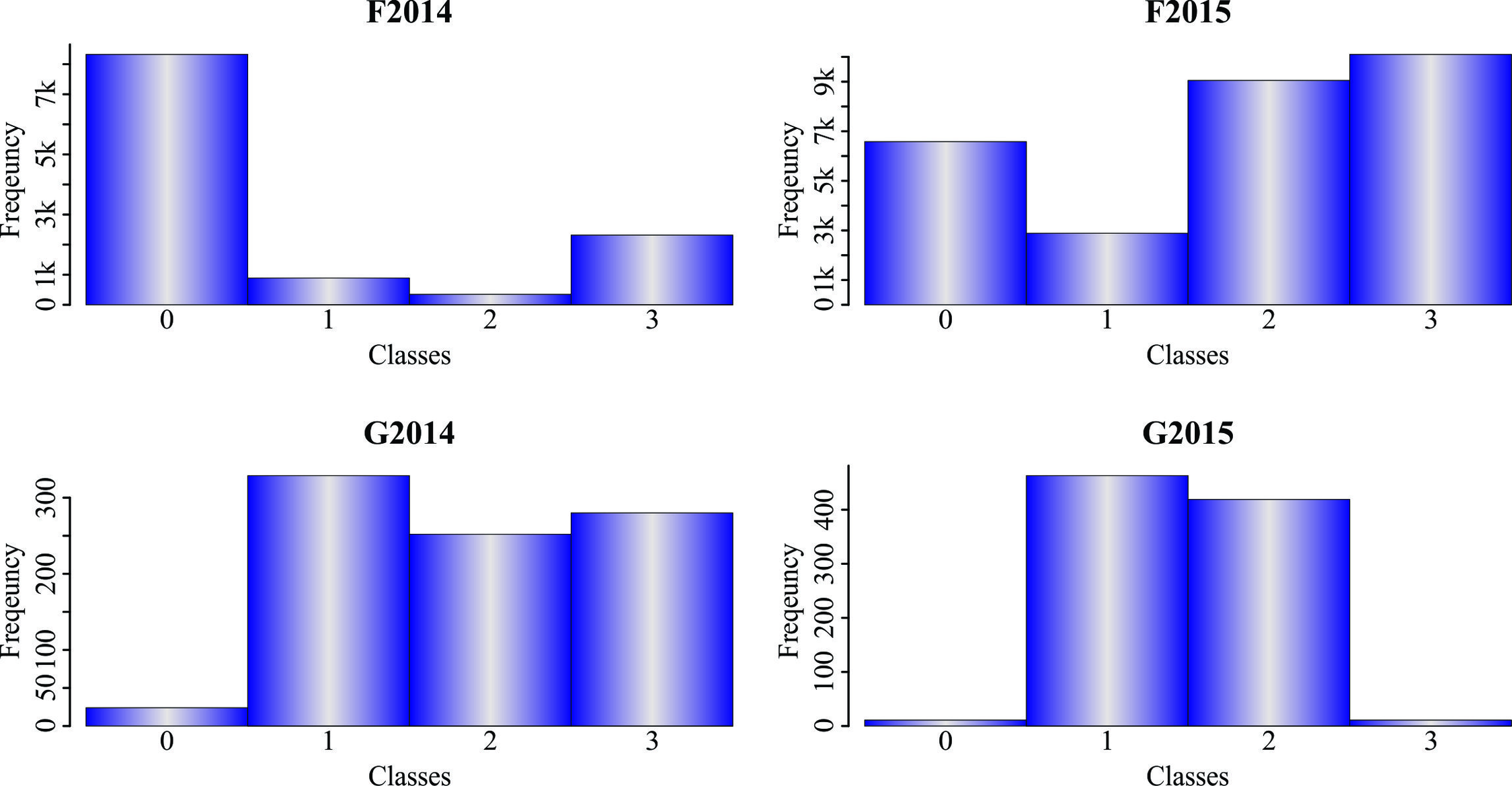

Supplement: S1 Fig — (TIF) [file pone.0179191.s001.tif]

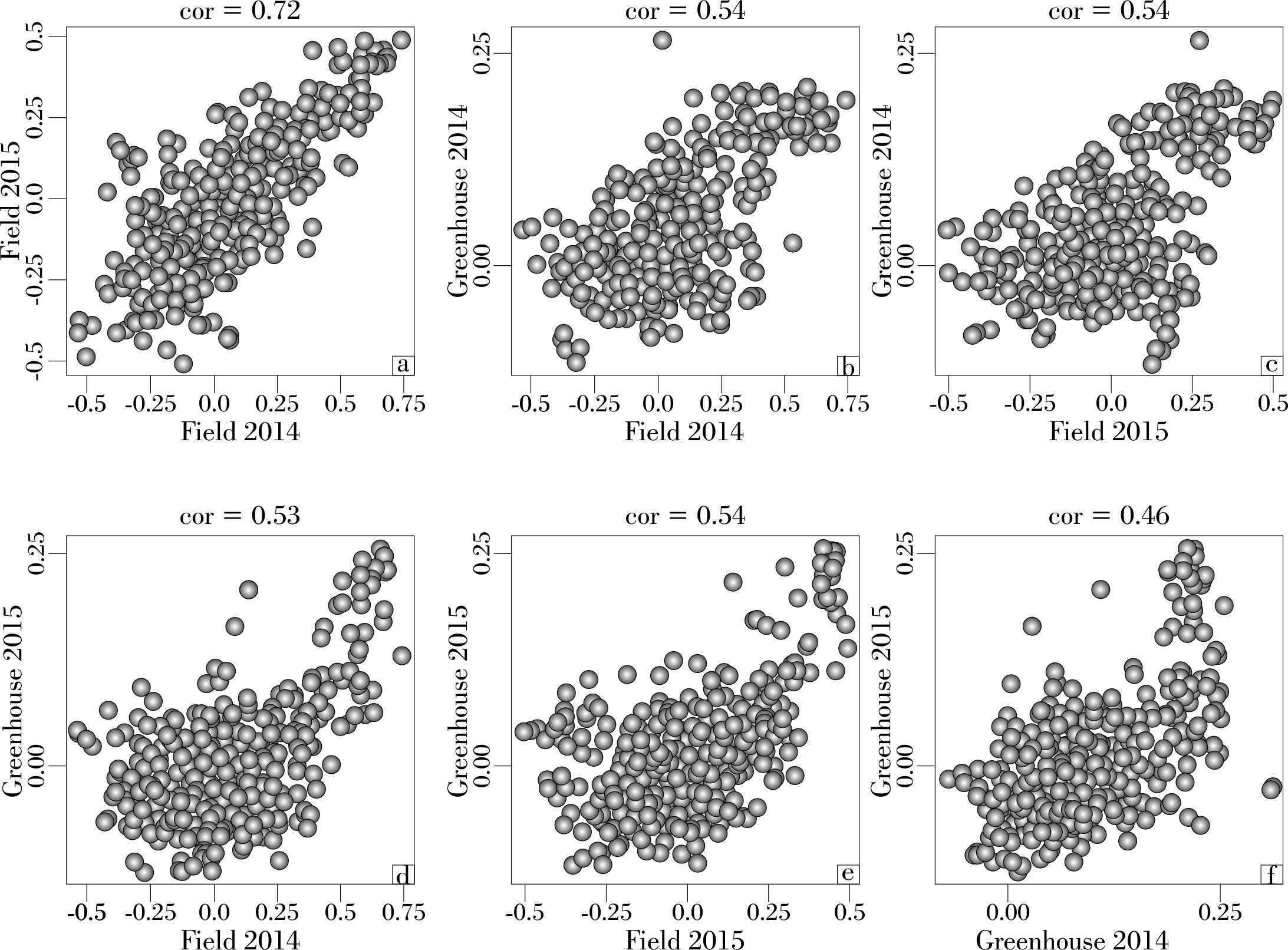

Supplement: S2 Fig — (TIF) [file pone.0179191.s002.tif]

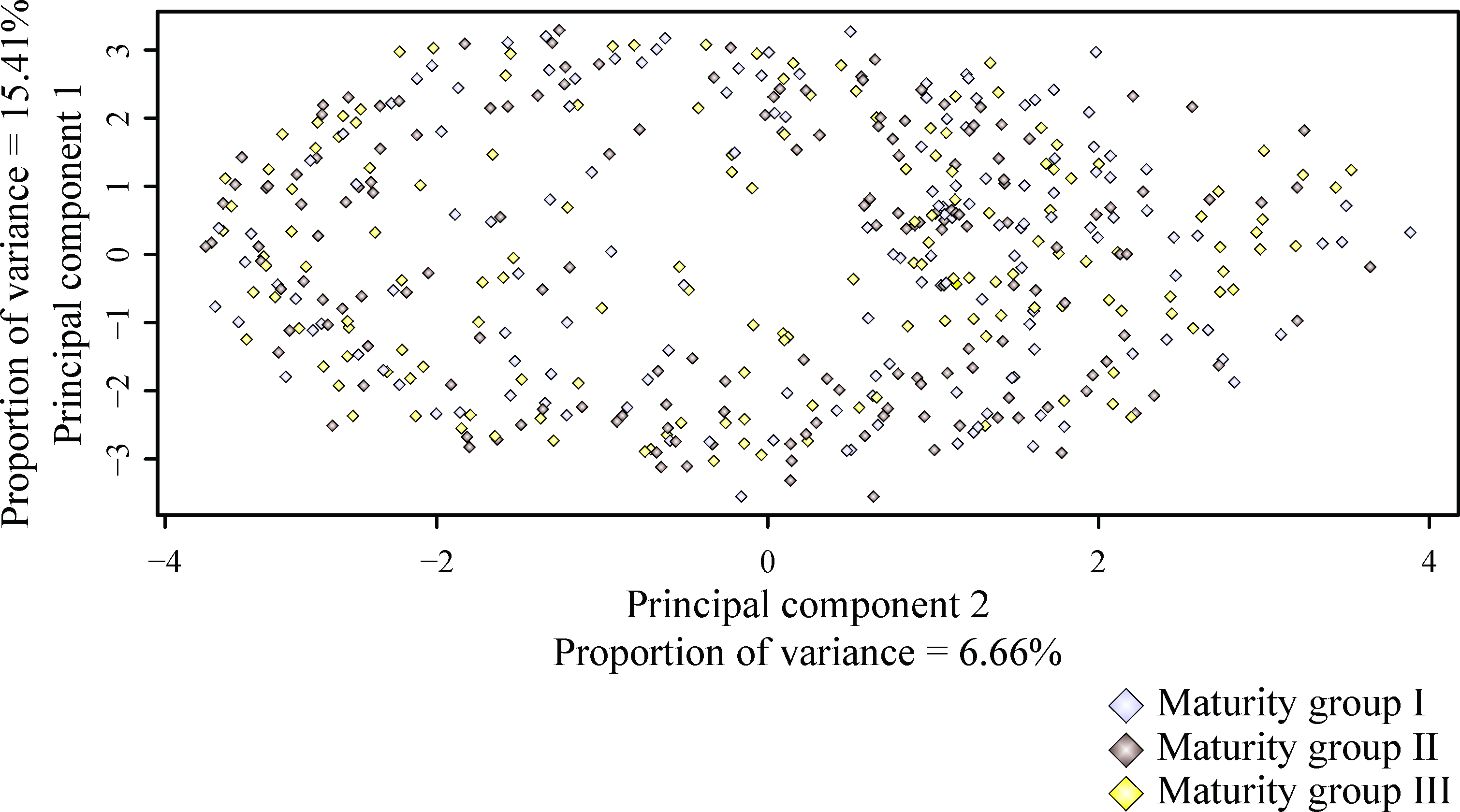

Supplement: S3 Fig — (TIF) [file pone.0179191.s003.tif]

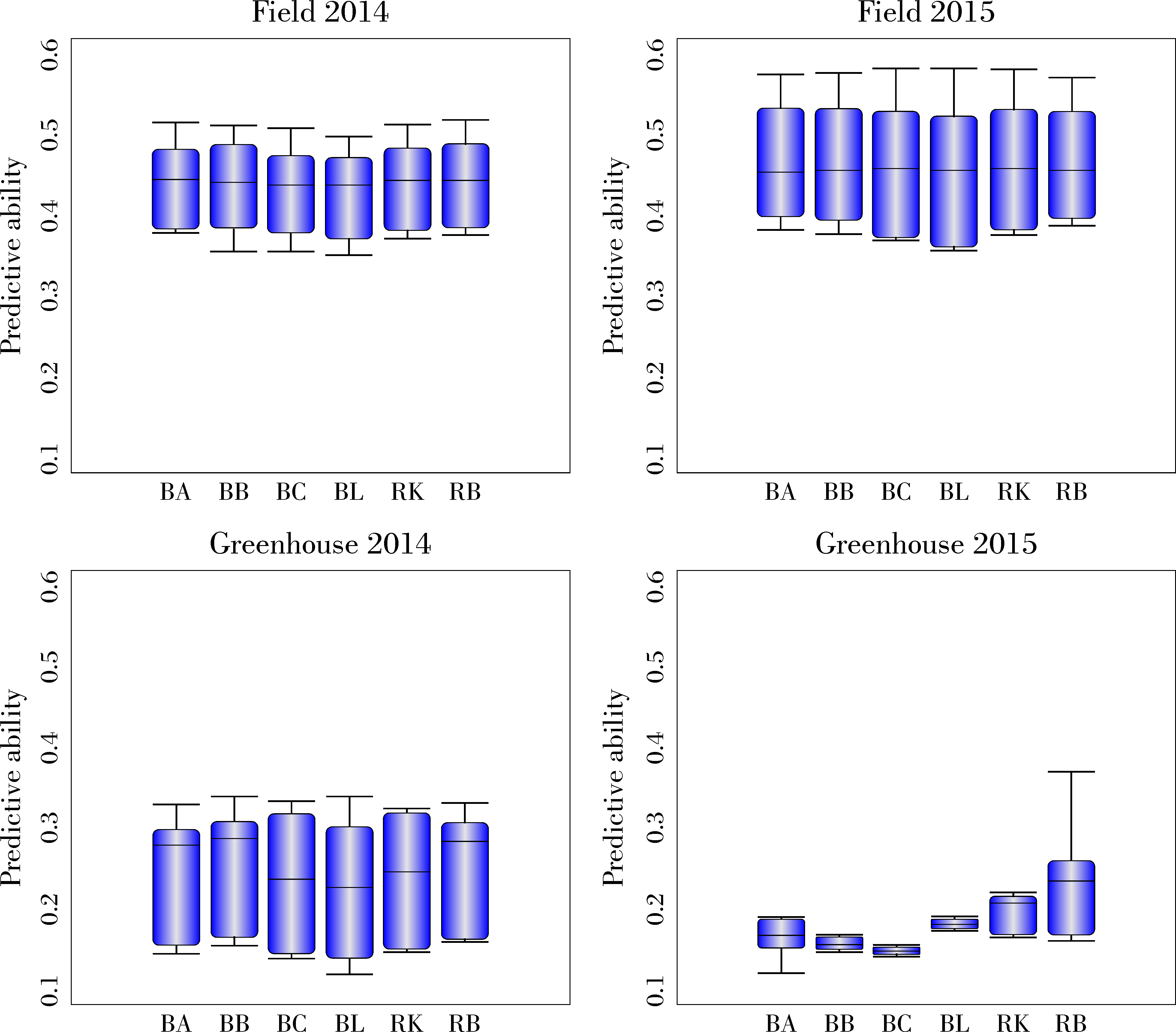

Supplement: S4 Fig — The range in predictive ability is among the 50 replicates of the cross validation experiment BA–Bayes A; BB–Bayes B; BC–Bayes Cπ; BL–Bayesian LASSO; GB–Genomic Best Linear Unbiased Prediction; RK–Reproducing Kernel Hilbert Space Regression; BR–Best Linear Unbiased Prediction. (TIF) [file pone.0179191.s004.tif]

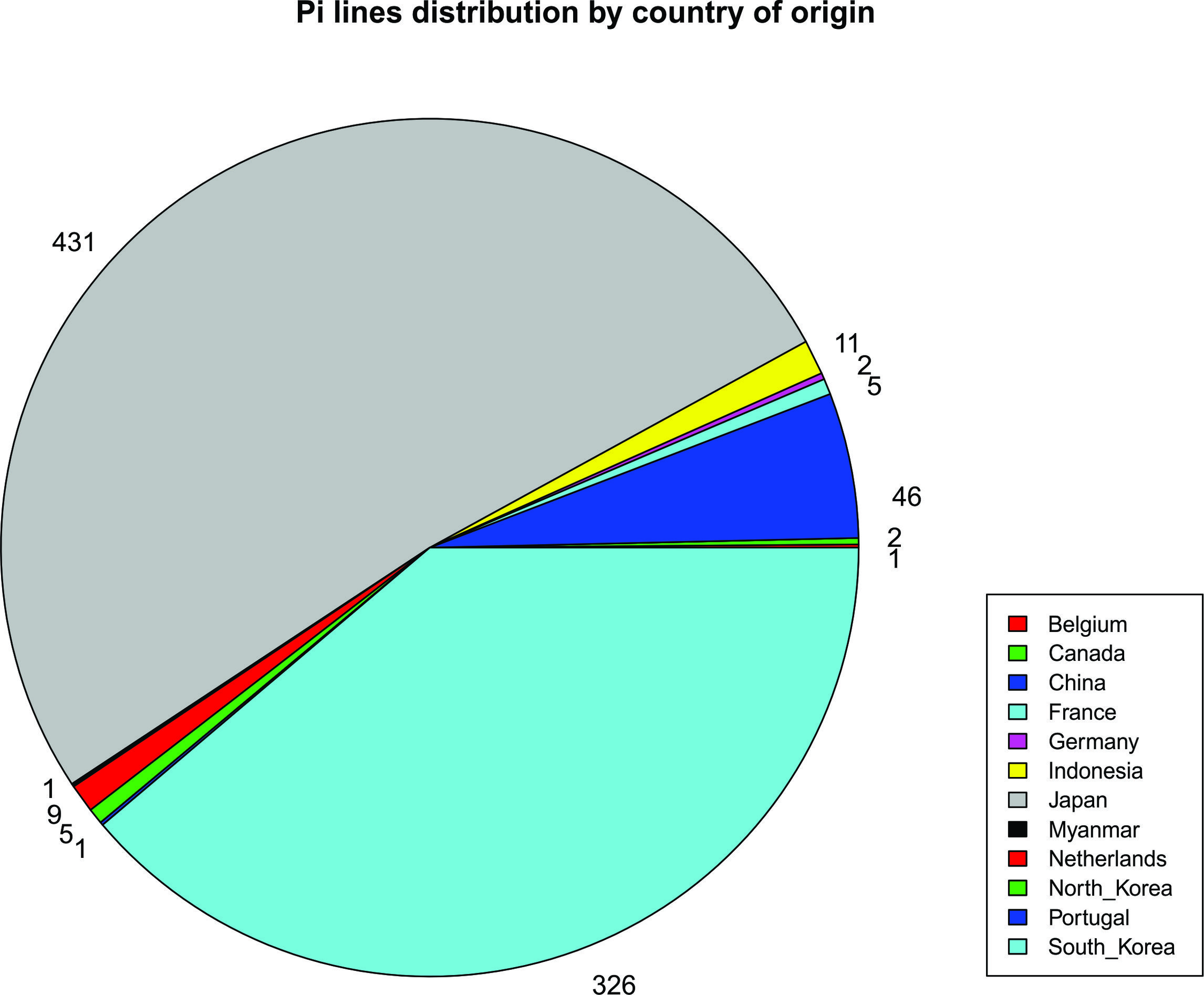

Supplement: S5 Fig — (TIF) [file pone.0179191.s005.tif]

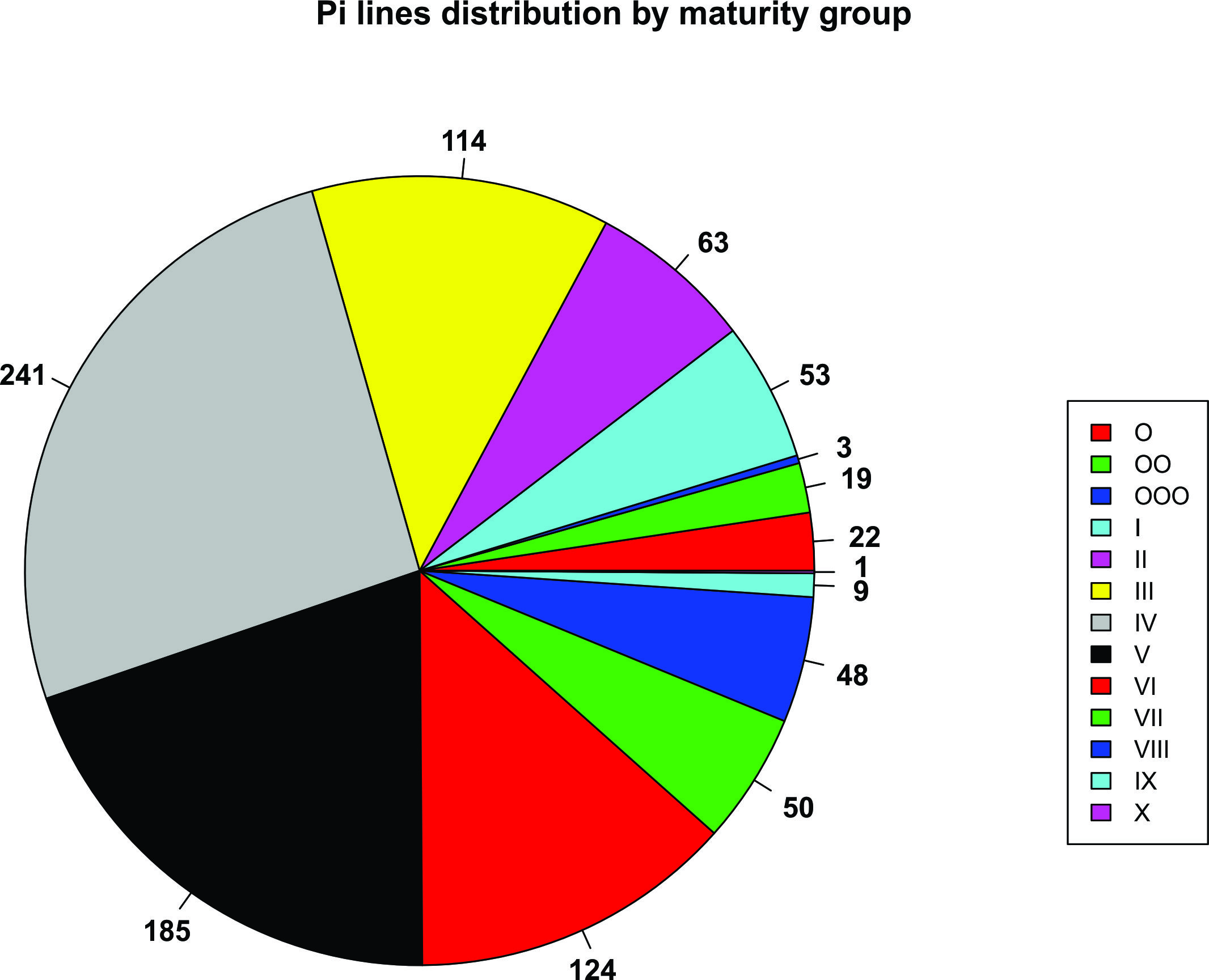

Supplement: S6 Fig — (TIF) [file pone.0179191.s006.tif]
