# Supplementary material for: Leveraging genomic prediction to scan germplasm collection for crop improvement
Source: PLoS One. 2017 Jun 9;12(6):e0179191. doi: 10.1371/journal.pone.0179191 (PMC5466325; doi:10.1371/journal.pone.0179191)
Supplement: S1 Table — (DOCX) [file pone.0179191.s007.docx]

**S1 Table**. 5% most resistant soybean accessions for the entire USDA soybean germplasm collection and their GEBVs.

| **PI** | **GEBV** | **PI** | **GEBV** | **PI** | **GEBV** | **PI** | **GEBV** |
| --- | --- | --- | --- | --- | --- | --- | --- |
| PI506718 | -0.70 | PI507139 | -0.61 | PI398912 | -0.59 | PI417144 | -0.58 |
| PI442019 | -0.69 | PI398822 | -0.61 | PI398727 | -0.59 | PI417435 | -0.58 |
| PI424339 | -0.69 | PI424242 | -0.61 | PI398637 | -0.59 | PI594280D | -0.58 |
| PI398311 | -0.69 | PI458276 | -0.61 | PI506664 | -0.59 | PI507449 | -0.58 |
| PI340017 | -0.69 | PI424590A | -0.61 | PI398859 | -0.59 | PI399106 | -0.58 |
| PI424353 | -0.69 | PI408307C | -0.61 | PI407903A | -0.59 | PI417451 | -0.58 |
| PI424368A | -0.68 | PI243549 | -0.61 | PI398957 | -0.59 | PI087165 | -0.58 |
| PI407907A | -0.68 | PI248515 | -0.61 | PI243517 | -0.59 | PI423899 | -0.58 |
| PI424348A | -0.68 | PI506822 | -0.61 | PI532466A | -0.59 | PI408140B | -0.58 |
| PI424373 | -0.68 | PI423776 | -0.61 | PI424247A | -0.59 | PI424276 | -0.58 |
| PI424358 | -0.68 | PI398693 | -0.61 | PI273483E | -0.59 | PI408052C | -0.58 |
| PI423826C | -0.68 | PI506775 | -0.61 | PI423908 | -0.59 | PI458290 | -0.58 |
| PI398857 | -0.68 | PI423777 | -0.61 | PI603702B | -0.59 | PI417201 | -0.58 |
| PI340005 | -0.68 | PI506547 | -0.61 | PI594695 | -0.59 | PI416953 | -0.58 |
| PI424351 | -0.68 | PI423772 | -0.61 | PI424425 | -0.59 | PI458244A | -0.58 |
| PI549028 | -0.68 | PI507540 | -0.61 | PI398639 | -0.59 | PI408199 | -0.58 |
| PI458123A | -0.68 | PI407758 | -0.61 | PI506488 | -0.59 | PI424570 | -0.58 |
| PI407877A | -0.68 | PI424549A | -0.61 | PI408168 | -0.59 | PI417142 | -0.58 |
| PI407972A | -0.68 | PI208782 | -0.61 | PI398280 | -0.59 | PI398765 | -0.58 |
| PI506706 | -0.68 | PI398244 | -0.61 | PI507260 | -0.59 | PI416858 | -0.58 |
| PI398930 | -0.68 | PI506987 | -0.61 | PI424290 | -0.59 | PI243521 | -0.58 |
| PI424143 | -0.68 | PI408135A | -0.61 | PI086128 | -0.59 | PI423907 | -0.58 |
| PI347563A | -0.68 | PI398392 | -0.61 | PI424263 | -0.59 | PI408048A | -0.58 |
| PI398953 | -0.68 | PI438274 | -0.61 | PI506919 | -0.59 | PI587878 | -0.58 |
| PI398747 | -0.68 | PI399031 | -0.61 | PI417132 | -0.59 | PI399047 | -0.58 |
| PI407939A | -0.68 | PI417466 | -0.61 | PI416870 | -0.59 | PI408161 | -0.58 |
| PI424152 | -0.67 | PI594198 | -0.61 | PI417018 | -0.59 | PI464879 | -0.58 |
| PI407998B | -0.67 | PI506810 | -0.61 | PI200531 | -0.59 | PI417431 | -0.58 |
| PI398290 | -0.67 | PI417561 | -0.61 | PI399126 | -0.59 | PI423844 | -0.58 |
| PI416809 | -0.67 | PI398978 | -0.61 | PI594267 | -0.59 | PI082555 | -0.58 |
| PI391588 | -0.67 | PI507084 | -0.61 | PI507448 | -0.59 | PI507227 | -0.58 |
| PI507436 | -0.67 | PI417434 | -0.61 | PI423885 | -0.59 | PI340053A | -0.58 |
| PI509079 | -0.67 | PI507273 | -0.61 | PI507305 | -0.59 | PI407964 | -0.58 |
| PI424350 | -0.67 | PI458197 | -0.61 | PI086028 | -0.59 | PI417125 | -0.58 |
| PI506869 | -0.67 | PI416800 | -0.61 | PI458040 | -0.59 | PI506776 | -0.58 |
| PI398707 | -0.66 | PI408307A | -0.61 | PI330635 | -0.59 | PI398870 | -0.58 |
| PI159924 | -0.66 | PI339985 | -0.61 | PI408052B | -0.59 | PI398872 | -0.58 |
| PI424466 | -0.66 | PI504498 | -0.61 | PI424302 | -0.59 | PI438278 | -0.58 |
| PI399042 | -0.66 | PI424156D | -0.61 | PI424366 | -0.59 | PI408290 | -0.58 |
| PI506714 | -0.66 | PI080847_2 | -0.61 | PI458117 | -0.59 | PI417307 | -0.58 |
| PI407979 | -0.66 | PI398876 | -0.61 | PI458189B | -0.59 | PI423969 | -0.58 |
| PI408055B | -0.65 | PI506691 | -0.61 | PI416778 | -0.59 | PI506768 | -0.58 |
| PI091719 | -0.65 | PI398503 | -0.61 | PI506771 | -0.59 | PI424435 | -0.58 |
| PI274420 | -0.65 | PI507073 | -0.61 | PI567036 | -0.59 | PI081037_2 | -0.58 |
| PI434980A | -0.65 | PI506720 | -0.61 | PI416759 | -0.59 | PI398561 | -0.58 |
| PI417266 | -0.65 | PI417469 | -0.61 | PI407852 | -0.59 | PI417158 | -0.58 |
| PI398399 | -0.65 | PI603404 | -0.61 | PI408315A | -0.59 | PI416792 | -0.58 |
| PI416981 | -0.65 | PI506773 | -0.61 | PI408312B | -0.59 | PI340020 | -0.58 |
| PI408121 | -0.65 | PI096354 | -0.61 | PI424550 | -0.59 | PI424407 | -0.58 |
| PI200453 | -0.65 | PI398306 | -0.61 | PI507229 | -0.59 | PI507428 | -0.58 |
| PI507216B | -0.65 | PI416996 | -0.61 | PI417111 | -0.59 | PI423769A | -0.58 |
| PI504496 | -0.65 | PI417202 | -0.61 | PI086543 | -0.59 | PI408076A | -0.58 |
| PI340039 | -0.65 | PI507250 | -0.61 | PI507056 | -0.59 | PI506774 | -0.58 |
| PI398315 | -0.65 | PI398648 | -0.61 | PI398989 | -0.59 | PI458059 | -0.58 |
| PI538403 | -0.65 | PI507078 | -0.61 | PI458111 | -0.59 | PI506815 | -0.58 |
| PI398964 | -0.65 | PI391581A | -0.61 | PI408207_1 | -0.59 | PI507457 | -0.58 |
| PI506707 | -0.65 | PI507514 | -0.61 | PI408028 | -0.59 | PI594219 | -0.58 |
| PI594021 | -0.65 | PI508296F | -0.61 | PI408075 | -0.59 | PI507493 | -0.58 |
| PI407927B | -0.65 | PI424377 | -0.61 | PI424382 | -0.59 | PI424293 | -0.58 |
| PI423870 | -0.65 | PI506907 | -0.61 | PI471904 | -0.59 | PI398913 | -0.58 |
| PI507214 | -0.65 | PI507356 | -0.61 | PI424229B | -0.59 | PI407975A | -0.58 |
| PI408019C | -0.64 | PI229342 | -0.61 | PI423798A | -0.59 | PI442008 | -0.58 |
| PI423745 | -0.64 | PI423814A | -0.61 | PI507311 | -0.59 | PI507362 | -0.58 |
| PI408307B | -0.64 | PI408109A | -0.61 | PI506870 | -0.59 | PI398651 | -0.58 |
| PI506796 | -0.64 | PI424423 | -0.61 | PI424605A | -0.59 | PI416937 | -0.58 |
| PI416945 | -0.64 | PI458245 | -0.60 | PI427137 | -0.59 | PI408315B | -0.58 |
| PI408308B | -0.64 | PI095853 | -0.60 | PI424467 | -0.59 | PI417317 | -0.58 |
| PI507211 | -0.64 | PI416754 | -0.60 | FC031684 | -0.59 | PI561391 | -0.58 |
| PI458141 | -0.64 | PI273483B | -0.60 | PI458307A | -0.59 | PI458278A | -0.58 |
| PI200548 | -0.64 | PI438295 | -0.60 | PI507285 | -0.59 | PI507045 | -0.58 |
| PI407861A | -0.64 | PI507539 | -0.60 | PI407933 | -0.59 | PI442005 | -0.58 |
| PI424456 | -0.64 | PI416884 | -0.60 | PI398934 | -0.59 | PI423838 | -0.58 |
| PI408154 | -0.64 | PI398987 | -0.60 | PI340047 | -0.59 | PI507011 | -0.58 |
| PI506499 | -0.64 | PI458230A | -0.60 | PI384469C | -0.59 | PI507063 | -0.58 |
| PI398713 | -0.64 | PI398708 | -0.60 | PI340033 | -0.59 | PI593948 | -0.58 |
| PI408308A | -0.64 | PI200487 | -0.60 | PI458175A | -0.59 | PI340007 | -0.58 |
| PI339995 | -0.64 | PI200503 | -0.60 | PI407952A | -0.59 | PI548587 | -0.58 |
| PI229346 | -0.64 | PI508296B | -0.60 | PI407850 | -0.59 | PI507415 | -0.58 |
| PI594247 | -0.64 | PI417393 | -0.60 | PI424134 | -0.59 | PI416951 | -0.58 |
| PI458295 | -0.64 | PI424161 | -0.60 | PI243545 | -0.59 | PI507132A | -0.58 |
| PI507292 | -0.64 | PI408024 | -0.60 | PI424337_1 | -0.59 | PI504493 | -0.58 |
| PI087606 | -0.64 | PI416812 | -0.60 | PI506763 | -0.59 | PI408332C | -0.58 |
| PI603167 | -0.64 | PI398726 | -0.60 | PI408285A | -0.59 | PI398650 | -0.58 |
| PI507576 | -0.64 | PI424270C | -0.60 | PI507067 | -0.59 | PI398684 | -0.58 |
| PI408309 | -0.64 | PI398763 | -0.60 | PI398654 | -0.59 | PI196161 | -0.58 |
| PI408040_2 | -0.64 | PI408197A | -0.60 | PI398661 | -0.59 | PI398772 | -0.58 |
| PI340052 | -0.64 | PI507563 | -0.60 | PI398415 | -0.59 | PI086452 | -0.58 |
| PI423889 | -0.64 | PI424549B | -0.60 | PI398814 | -0.59 | PI227222 | -0.58 |
| PI238931 | -0.64 | PI398759 | -0.60 | PI205085 | -0.59 | PI506549 | -0.58 |
| PI398351 | -0.64 | FC019979_1 | -0.60 | PI458101 | -0.59 | PI603651 | -0.58 |
| **PI281850** | -0.64 | PI398336 | -0.60 | PI227565 | -0.59 | PI181571 | -0.58 |
| PI507280 | -0.64 | PI507026 | -0.60 | PI417205 | -0.59 | PI085420_1 | -0.58 |
| PI224275 | -0.64 | PI603154 | -0.60 | PI506631 | -0.59 | PI508294 | -0.58 |
| PI398700 | -0.63 | PI424239 | -0.60 | PI087047 | -0.59 | PI458259 | -0.58 |
| PI227563 | -0.63 | PI408131B | -0.60 | PI507566 | -0.59 | PI082312N | -0.58 |
| PI458291 | -0.63 | PI506592 | -0.60 | PI398504 | -0.59 | PI458198 | -0.58 |
| PI423896 | -0.63 | PI458113 | -0.60 | PI398600 | -0.59 | PI196171 | -0.58 |
| PI506740 | -0.63 | PI506867 | -0.60 | PI507130 | -0.59 | PI417246 | -0.58 |
| PI424159A | -0.63 | PI157424 | -0.60 | PI274206 | -0.59 | PI398521 | -0.58 |
| PI507032 | -0.63 | PI507492 | -0.60 | PI417260B | -0.59 | PI398484 | -0.58 |
| PI086007 | -0.63 | PI398585 | -0.60 | PI082558 | -0.59 | PI548419 | -0.58 |
| PI507171 | -0.63 | PI417218 | -0.60 | PI506576 | -0.59 | PI339988 | -0.58 |
| PI088816 | -0.63 | PI506915 | -0.60 | PI398571 | -0.59 | PI398890 | -0.58 |
| PI399020 | -0.63 | PI424356 | -0.60 | PI507255 | -0.59 | PI398686 | -0.58 |
| PI506494 | -0.63 | PI398662 | -0.60 | PI507530 | -0.59 | PI200470 | -0.58 |
| PI417430 | -0.63 | PI398584 | -0.60 | PI398927 | -0.59 | PI408327A | -0.58 |
| PI243551 | -0.63 | PI613559A | -0.60 | PI506811 | -0.59 | PI507039 | -0.58 |
| PI424616 | -0.63 | PI507571 | -0.60 | PI342003 | -0.59 | PI506744 | -0.58 |
| PI507460 | -0.63 | PI417470 | -0.60 | PI507100 | -0.59 | PI285089 | -0.58 |
| PI507240 | -0.63 | PI398230 | -0.60 | PI407946_2 | -0.59 | FC003981 | -0.58 |
| PI417079 | -0.63 | PI398660 | -0.60 | PI592906 | -0.59 | PI506877 | -0.58 |
| PI507360 | -0.63 | PI507390 | -0.60 | PI398794 | -0.59 | PI340031A | -0.58 |
| PI399027 | -0.63 | PI424270A | -0.60 | PI427138 | -0.59 | PI507380 | -0.58 |
| PI087457 | -0.63 | PI398751 | -0.60 | PI417413 | -0.59 | PI407925 | -0.58 |
| PI417195 | -0.63 | PI424318 | -0.60 | PI458261 | -0.59 | PI603916 | -0.58 |
| PI398440 | -0.63 | PI085009_1 | -0.60 | PI458091 | -0.59 | PI423917 | -0.58 |
| PI339980 | -0.63 | PI086510 | -0.60 | PI084970 | -0.59 | PI506777 | -0.58 |
| PI507397 | -0.63 | PI407761 | -0.60 | PI398925 | -0.59 | PI399090 | -0.58 |
| PI083893 | -0.63 | PI398720 | -0.60 | PI424222B | -0.59 | PI507216A | -0.58 |
| PI507125 | -0.63 | PI398716 | -0.60 | PI407926B | -0.59 | PI506946 | -0.58 |
| PI398297 | -0.63 | PI509107 | -0.60 | PI507426 | -0.59 | PI424489A | -0.58 |
| PI224272 | -0.63 | PI398820 | -0.60 | PI507532 | -0.59 | PI423931 | -0.58 |
| PI458244C | -0.63 | PI424563 | -0.60 | PI093055 | -0.59 | PI603908 | -0.58 |
| PI507333 | -0.63 | PI458300 | -0.60 | PI408222A | -0.59 | PI423851 | -0.58 |
| PI342437 | -0.63 | PI398417 | -0.60 | PI398908 | -0.59 | PI398667 | -0.58 |
| PI458297 | -0.63 | PI416989 | -0.60 | PI507121 | -0.59 | PI507434 | -0.58 |
| PI506902 | -0.63 | PI081041_1 | -0.60 | PI398832 | -0.59 | PI507261 | -0.58 |
| PI408022 | -0.63 | PI506906 | -0.60 | PI339997 | -0.59 | PI507092 | -0.58 |
| PI416978 | -0.63 | PI506603 | -0.60 | PI096978 | -0.59 | PI399104 | -0.58 |
| PI340053B | -0.63 | PI603742D | -0.60 | PI507265 | -0.59 | PI261472 | -0.58 |
| PI398665 | -0.63 | PI398254 | -0.60 | PI593648 | -0.59 | PI398235 | -0.58 |
| PI506532 | -0.63 | PI398497 | -0.60 | PI506724 | -0.59 | PI506569 | -0.58 |
| PI417019 | -0.63 | PI603416 | -0.60 | PI507220 | -0.59 | PI408071 | -0.58 |
| PI398911 | -0.63 | PI424281A | -0.60 | PI458307B | -0.59 | PI157469 | -0.58 |
| PI424535A | -0.63 | PI424166 | -0.60 | PI423892 | -0.59 | PI408099 | -0.58 |
| PI340001 | -0.63 | PI398439 | -0.60 | PI398533 | -0.59 | PI398559 | -0.58 |
| PI408067A | -0.63 | PI145079 | -0.60 | PI424359 | -0.59 | PI424422 | -0.58 |
| PI504490 | -0.63 | PI507491 | -0.60 | PI424319 | -0.59 | PI458250 | -0.58 |
| PI340048 | -0.63 | PI507431 | -0.60 | PI458049 | -0.59 | PI507456 | -0.58 |
| PI406708 | -0.63 | PI398903 | -0.60 | PI458178 | -0.59 | PI407801 | -0.58 |
| PI423746 | -0.63 | PI548331 | -0.60 | PI506645 | -0.59 | PI424272B | -0.58 |
| PI506766 | -0.63 | PI408220 | -0.60 | PI506761 | -0.59 | PI424494 | -0.58 |
| PI360834 | -0.63 | PI398529 | -0.60 | PI458237 | -0.59 | PI408334 | -0.58 |
| PI084949 | -0.63 | PI407875A | -0.60 | PI407755 | -0.59 | PI086071 | -0.58 |
| PI408248A | -0.63 | PI416777 | -0.60 | PI507288 | -0.59 | PI157477 | -0.58 |
| PI399039 | -0.63 | PI507425 | -0.60 | PI423799B | -0.59 | PI587851C | -0.58 |
| PI398441 | -0.63 | PI398712 | -0.60 | PI458132 | -0.59 | PI548323 | -0.58 |
| PI398437 | -0.63 | PI458123B | -0.60 | PI548327 | -0.59 | PI423792 | -0.58 |
| PI458294 | -0.63 | PI603743A | -0.60 | PI408005 | -0.59 | PI408098 | -0.58 |
| PI398821 | -0.63 | PI423801 | -0.60 | PI458242 | -0.59 | PI486354A | -0.58 |
| PI408018 | -0.62 | PI391585 | -0.60 | PI398914 | -0.59 | PI524993 | -0.58 |
| PI408318A | -0.62 | PI398312 | -0.60 | PI587636 | -0.59 | PI507497 | -0.58 |
| PI157454 | -0.62 | PI561360 | -0.60 | PI398758 | -0.58 | PI423953 | -0.58 |
| PI246365 | -0.62 | **PI549066** | -0.60 | PI407875B | -0.58 | PI424376 | -0.58 |
| PI408226A | -0.62 | PI507437 | -0.60 | PI424149 | -0.58 | PI200450 | -0.58 |
| PI339981 | -0.62 | PI506723 | -0.60 | PI458189A | -0.58 | PI458050 | -0.58 |
| PI504497 | -0.62 | PI196148 | -0.60 | PI408275 | -0.58 | PI417129B | -0.58 |
| PI408131A | -0.62 | PI506753 | -0.60 | PI229330 | -0.58 | PI458061A | -0.58 |
| PI548301 | -0.62 | PI398638 | -0.60 | PI407849 | -0.58 | PI423815 | -0.58 |
| PI408198 | -0.62 | PI408224B | -0.60 | PI408167C | -0.58 | PI423790 | -0.58 |
| PI082210 | -0.62 | PI407926A | -0.60 | PI423954 | -0.58 | PI398653 | -0.58 |
| PI408212A | -0.62 | PI398749 | -0.60 | PI458051B | -0.58 | PI398285 | -0.58 |
| PI157428 | -0.62 | PI458042 | -0.60 | PI096786_1 | -0.58 | PI086060 | -0.58 |
| PI398581 | -0.62 | PI398805 | -0.60 | PI507282 | -0.58 | PI085437 | -0.58 |
| PI157410 | -0.62 | PI092707S | -0.60 | PI398482 | -0.58 | PI398640 | -0.58 |
| PI423877 | -0.62 | PI340018 | -0.60 | PI200535 | -0.58 | PI399001 | -0.58 |
| PI416920 | -0.62 | PI248511 | -0.60 | PI423944 | -0.58 | PI080459 | -0.58 |
| PI398190 | -0.62 | PI424379 | -0.60 | PI180524 | -0.58 | PI506767 | -0.58 |
| PI408089 | -0.62 | PI424352 | -0.60 | FC019979_5 | -0.58 | PI507248 | -0.58 |
| PI399005 | -0.62 | PI416903 | -0.60 | PI407877B | -0.58 | PI458140 | -0.58 |
| PI408090 | -0.62 | PI407928 | -0.60 | PI458160 | -0.58 | PI416810 | -0.58 |
| PI424142 | -0.62 | PI424408 | -0.60 | PI229337 | -0.58 | PI423805 | -0.58 |
| PI398265 | -0.62 | PI424170 | -0.60 | PI548342 | -0.58 | PI417234 | -0.58 |
| PI398990 | -0.62 | PI083892 | -0.60 | PI086022 | -0.58 | PI408242 | -0.58 |
| PI082315 | -0.62 | PI398505 | -0.60 | PI417287 | -0.58 | PI506752 | -0.58 |
| PI458086 | -0.62 | PI507361 | -0.60 | PI407903B | -0.58 | PI442010 | -0.58 |
| PI423890A | -0.62 | PI424154A | -0.60 | PI507357 | -0.58 | PI548353 | -0.58 |
| PI157439 | -0.62 | PI398599 | -0.60 | PI203404 | -0.58 | PI417153 | -0.57 |
| PI603742A | -0.62 | PI417147 | -0.60 | PI416956 | -0.58 | PI229313 | -0.57 |
| PI407946_1 | -0.62 | PI506937 | -0.60 | PI416769B | -0.58 | PI085559 | -0.57 |
| PI603911A | -0.62 | PI567039 | -0.59 | PI398685 | -0.58 | PI424384 | -0.57 |
| PI458138 | -0.62 | PI229359 | -0.59 | PI398885 | -0.58 | PI416952 | -0.57 |
| PI157447 | -0.62 | PI506837 | -0.59 | PI423965 | -0.58 | PI417472B | -0.57 |
| PI424605B | -0.62 | PI458238 | -0.59 | PI205092 | -0.58 | PI416839 | -0.57 |
| PI408186C | -0.62 | PI416766 | -0.59 | PI424568 | -0.58 | PI506854 | -0.57 |
| PI095801 | -0.62 | PI407922 | -0.59 | PI424488A | -0.58 | PI423948A | -0.57 |
| PI398711 | -0.62 | PI398298 | -0.59 | PI424540 | -0.58 | PI507279 | -0.57 |
| PI506835 | -0.62 | PI398313 | -0.59 | PI339993 | -0.58 | PI424329 | -0.57 |
| PI423843 | -0.62 | PI507179 | -0.59 | PI507515 | -0.58 | PI506677 | -0.57 |
| PI416760 | -0.62 | PI506704 | -0.59 | PI506502 | -0.58 | PI417414B | -0.57 |
| PI567273A | -0.62 | PI423873 | -0.59 | PI506730 | -0.58 | PI506585B | -0.57 |
| PI416913 | -0.62 | PI417316 | -0.59 | PI506698 | -0.58 | PI548350 | -0.57 |
| PI408178 | -0.62 | PI507496 | -0.59 | PI408025 | -0.58 | PI424211 | -0.57 |
| PI082218 | -0.62 | PI507079 | -0.59 | PI085625 | -0.58 | PI407851 | -0.57 |
| PI506903 | -0.62 | PI423890C | -0.59 | PI458205 | -0.58 | PI423720 | -0.57 |
| PI594286 | -0.62 | PI407870 | -0.59 | PI507455 | -0.58 | PI458061B | -0.57 |
| PI399063 | -0.62 | PI416947 | -0.59 | PI417447 | -0.58 | PI342438 | -0.57 |
| PI091734 | -0.62 | PI398576 | -0.59 | PI229326 | -0.58 | PI090573 | -0.57 |
| PI407855 | -0.62 | PI407894 | -0.59 | PI417488 | -0.58 | PI408026 | -0.57 |
| PI518833 | -0.62 | PI417333 | -0.59 | PI518295 | -0.58 | PI507210 | -0.57 |
| PI416788 | -0.62 | PI069512 | -0.59 | PI424555B | -0.58 | PI486354B | -0.57 |
| PI408184B | -0.62 | PI458248 | -0.59 | PI417281 | -0.58 | PI509089 | -0.57 |
| PI404163 | -0.62 | PI458240 | -0.59 | PI226588 | -0.58 | PI408128 | -0.57 |
| PI507441A | -0.62 | PI398994 | -0.59 | PI416904B | -0.58 | PI605864 | -0.57 |
| PI398709 | -0.62 | PI597411B | -0.59 | PI506888 | -0.58 | PI424495 | -0.57 |
| PI507099 | -0.62 | PI458137 | -0.59 | PI423744 | -0.58 | PI548352 | -0.57 |
| PI424140 | -0.62 | PI398806 | -0.59 | PI084992 | -0.58 | PI340026 | -0.57 |
| PI509110B | -0.62 | PI424261 | -0.59 | PI187155 | -0.58 | PI548441 | -0.57 |
| PI424558B | -0.62 | PI416846 | -0.59 | PI548435 | -0.58 | PI417456 | -0.57 |
| PI506832 | -0.62 | PI506758 | -0.59 | PI398361 | -0.58 | PI417472D | -0.57 |
| PI458176 | -0.62 | PI506663 | -0.59 | PI507104 | -0.58 | PI506784 | -0.57 |
| PI507095B | -0.62 | PI399017 | -0.59 | PI084979 | -0.58 | PI458256 | -0.57 |
| PI399074 | -0.62 | PI567054C | -0.59 | PI416967 | -0.58 | PI398226 | -0.57 |
| PI080845_1 | -0.62 | PI416969 | -0.59 | PI407817 | -0.58 | PI507226A | -0.57 |
| PI506597 | -0.62 | PI423893 | -0.59 | PI274423 | -0.58 | PI507440 | -0.57 |
| PI594268A | -0.62 | PI424297 | -0.59 | PI507320B | -0.58 | PI423983 | -0.57 |
| PI417425 | -0.62 | PI507050 | -0.59 | PI532473 | -0.58 | PI549055 | -0.57 |
| PI424148 | -0.62 | PI408051 | -0.59 | PI261469 | -0.58 | PI567279A | -0.57 |
| PI417225 | -0.62 | PI417034 | -0.59 | PI507331 | -0.58 | PI417421 | -0.57 |
| PI506729 | -0.62 | PI227560 | -0.59 | PI423829 | -0.58 | PI507554 | -0.57 |
| PI423826B | -0.62 | PI434980B | -0.59 | PI548416 | -0.58 | PI416966 | -0.57 |
| PI424492 | -0.62 | PI424277B | -0.59 | PI423890B | -0.58 | PI507075 | -0.57 |
| PI398902 | -0.62 | PI339991 | -0.59 | PI506689 | -0.58 | PI424381 | -0.57 |
| PI424169B | -0.62 | PI339977 | -0.59 | PI612720B | -0.58 | PI548661 | -0.57 |
| PI398260 | -0.62 | PI424444A | -0.59 | PI424411 | -0.58 | PI407949 | -0.57 |
| PI507031 | -0.62 | PI398422 | -0.59 | PI088353 | -0.58 | PI059849 | -0.57 |
| PI507441B | -0.62 | PI506830 | -0.59 | PI507106 | -0.58 | PI423762 | -0.57 |
| PI507485 | -0.62 | PI408156 | -0.59 | PI398831 | -0.58 | PI339986 | -0.57 |
| PI603165B | -0.62 | PI438294 | -0.59 | PI088490_1 | -0.58 | PI458065B | -0.57 |
| PI506719 | -0.62 | PI398641 | -0.59 | PI408270A | -0.58 | PI507049 | -0.57 |
| PI594289 | -0.61 | PI437733 | -0.59 | PI398680 | -0.58 | PI416943 | -0.57 |
| PI506593 | -0.61 | PI417436 | -0.59 | PI417026 | -0.58 | PI407998A | -0.57 |
| PI200534 | -0.61 | PI423943 | -0.59 | PI408321 | -0.58 | PI567279B | -0.57 |
| PI424558A | -0.61 | PI507442B | -0.59 | PI408226B | -0.58 | PI506598 | -0.57 |
| PI507438 | -0.61 | PI424150 | -0.59 | PI416998 | -0.58 | PI408336 | -0.57 |
| PI416770 | -0.61 | PI430737 | -0.59 | PI408130 | -0.58 | PI458175D | -0.57 |
| PI424513 | -0.61 | PI173994 | -0.59 | PI506681 | -0.58 | PI200546 | -0.57 |
| PI509088 | -0.61 | PI587925 | -0.59 | PI458135 | -0.58 | PI417114 | -0.57 |
| PI416925 | -0.61 | PI507513 | -0.59 | PI458255 | -0.58 | PI507291 | -0.57 |
| PI408019A | -0.61 | PI458246A | -0.59 | PI417194 | -0.58 | PI408066B | -0.57 |
| PI506505 | -0.61 | PI603161 | -0.59 | PI417159 | -0.58 | PI398407 | -0.57 |
| PI090576_2 | -0.61 | PI398406 | -0.59 | PI398939 | -0.58 | PI424255B | -0.57 |
| PI398766 | -0.61 | PI423923 | -0.59 | PI506866 | -0.58 | PI417126 | -0.57 |
| PI445831 | -0.61 | PI398397 | -0.59 | PI424337_2 | -0.58 | PI567538B | -0.57 |
| PI095769 | -0.61 | PI200493 | -0.59 | PI417176 | -0.58 | PI506741 | -0.57 |
| PI417405 | -0.61 | PI507290 | -0.59 | PI398349 | -0.58 | PI417144 | -0.58 |
